# Suggestive Evidence for Causal Effect of Leptin Levels on Risk for Anorexia Nervosa: Results of a Mendelian Randomization Study

**DOI:** 10.3389/fgene.2021.733606

**Published:** 2021-09-14

**Authors:** Triinu Peters, Jochen Antel, Roaa Naaresh, Björn-Hergen Laabs, Manuel Föcker, Nicola Albers, Judith Bühlmeier, Anke Hinney, Lars Libuda, Johannes Hebebrand

**Affiliations:** ^1^ Department of Child and Adolescent Psychiatry, Psychosomatics and Psychotherapy, University Hospital Essen, University of Duisburg-Essen, Essen, Germany; ^2^ Institut für Medizinische Biometrie und Statistik, Universität zu Lübeck, Universitätsklinikum Schleswig-Holstein, Lübeck, Germany; ^3^ Department of Child and Adolescent Psychiatry, University of Münster, Münster, Germany; ^4^ Institute of Nutrition, Consumption and Health, Faculty of Natural Sciences, Paderborn University, Paderborn, Germany; ^5^ Evangelisches Krankenhaus Düsseldorf, Children’s Hospital, Düsseldorf, Germany

**Keywords:** leptin levels, anorexia nervosa, mendelian randomization (MR), body mass index, hypoleptinemia

## Abstract

Genetic correlations suggest a coexisting genetic predisposition to both low leptin levels and risk for anorexia nervosa (AN). To investigate the causality and direction of these associations, we performed bidirectional two-sample Mendelian randomization (MR) analyses using data of the most recent genome-wide association study (GWAS) for AN and both a GWAS and an exome-wide-association-study (EWAS) for leptin levels. Most MR methods with genetic instruments from GWAS showed a causal effect of lower leptin levels on higher risk of AN (e.g. IVW b = −0.923, *p* = 1.5 × 10^−4^). Because most patients with AN are female, we additionally performed analyses using leptin GWAS data of females only. Again, there was a significant effect of leptin levels on the risk of AN (e.g. IVW b = −0.826, *p* = 1.1 × 10^−04^). MR with genetic instruments from EWAS showed no overall effect of leptin levels on the risk for AN. For the opposite direction, MR revealed no causal effect of AN on leptin levels. If our results are confirmed in extended GWAS data sets, a low endogenous leptin synthesis represents a risk factor for developing AN.

## Introduction

Leptin plays an essential role in the physiology and pathophysiology of energy homeostasis, endocrinology and metabolism (Berthoud, 2011; Mantzoros et al., 2011; Peelman et al., 2014). The hormone is involved in hedonic hunger and satiety circuits (Berthoud, 2011; Hinney et al., 2014) and in eating behavior (Farooqi et al., 2007). *Via* changing the sensitivity of brain reward circuits to food stimuli (Volkow et al., 2011), leptin has been shown to influence eating behavior and to modulate food intake (Miller, 2011; Hebebrand et al., 2014; Miller et al., 2014; Fernandes et al., 2015; Benbaibeche et al., 2021). The mesoaccumbal dopamine pathway seemingly acts as a direct target for leptin (Egecioglu et al., 2011) and confers incentive motivation for natural rewards like food. Leptin additionally influences emotions, cognition, and behavior, particularly in starvation (Lu, 2007; Morrison, 2009; Higgs et al., 2017; Hebebrand et al., 2019; Zou et al., 2019; Milos et al., 2020; Antel et al., 2021). Leptin is mainly synthesized in adipocytes, but is also expressed in several other tissues. Leptin binds to leptin receptors which are located in several brain regions and peripheral tissues (Margetic et al., 2002).

In this study, we focus on leptin levels in anorexia nervosa (AN). AN is a severe psychiatric disorder characterized by underweight and hypoleptinemia in addition to AN specific cognitions and emotions (Hebebrand et al., 1995; Grinspoon et al., 1996; Hebebrand et al., 1997; Hebebrand and Bulik, 2011; American Psychiatric Association, 2013; Misra and Klibanski, 2014). Our study was stimulated by recent case reports suggesting pronounced effects of recombinant human leptin on such cognitions and emotions (Milos et al., 2020; Antel et al., 2021).

Based on recent genetic findings, AN has been defined as a metabo-psychiatric disorder (Watson et al., 2019). This hypothesis is based on genetic correlations to a range of anthropometric and metabolic phenotypes. Thus, Watson et al. (2019) showed inverse genetic correlations between the risk for AN and levels of leptin, fasting insulin, body mass index (BMI), body fat percentage, risk of obesity, insulin resistance, and type 2 diabetes; a positive correlation was observed with levels of high density lipoproteins (HDL). However, not all genetic correlations, including those with leptin levels and type 2 diabetes, remained significant after adjusting for BMI (Watson et al., 2019). The negative genetic correlations between body fat percentage and AN were stronger in women than in men (Hübel et al., 2019).

To evaluate causality and direction of leptin levels for the risk of AN, Mendelian Randomization (MR) is the appropriate approach (Davey Smith and Ebrahim, 2003; Burgess et al., 2015; Zhao et al., 2021). Mendelian randomization uses genetic data and the assumption of a random transmission of genetic information at conception to the next generation with a random distribution of covariates. MR is therefore a kind of a randomized control study (RCT) with quasi-randomization (Ebrahim and Davey Smith, 2008; Lawlor et al., 2008). The basic assumption of MR is that there are genetic variants that alter the level of a modifiable environmental exposure or mirror the biological effects of that environmental exposure and that this modifiable environmental exposure alters the risk of a specific disease. Polymorphisms can be markers for such variants with a biological function (Davey Smith and Ebrahim, 2008). Common genetic polymorphisms identified to be relevant for the environmental exposure of interest e.g. in genome-wide association studies (GWAS) can be used as proxies for the exposure whose measurement is impossible, inaccurate or confounded. In this way it is possible to investigate the effect of this exposure on disease risk excluding the risk of residual confounding. Since the genetic variants cannot be altered by the disease or the environment, reverse causality is ruled out in an MR study, as there is no evidence for selection bias in which participants enter into the genetic studies on which MR is based (Davey Smith and Ebrahim, 2008). Additional advantages of MR over RCTs or longitudinal observational studies are that practical issues (such as high costs and long study duration) or ethical problems can be neglected (König and Greco, 2018). In MR, genetic variants are equivalent to lifetime differences in exposure and indicate the long-term effects on disease (outcome). Accordingly, they generate more realistic estimates of causal effects, because they are free of measurement error or short-term variations in exposure (Davey Smith and Ebrahim, 2005).

The aim of this study was to investigate whether there is a causal relationship between leptin levels and AN. We performed a two-sample MR which uses two different study samples for the risk factor and the outcome phenotype as data (Pierce and Burgess, 2013). Corresponding reverse MR analyses were conducted to test for opposite causality. We ran analyses based on both leptin levels unadjusted and adjusted for BMI and in data sets based on both sexes combined. However, we a priori considered the analysis based on leptin levels in females as the most valid. The reasons include our intention to attempt to eliminate effects of the well-known association between BMI and leptin levels and to focus on leptin secretion in females. Thus, both a higher percent body fat and increased synthesis of leptin per unit of fat mass potentially explain why females have higher circulating leptin levels than males (Hellström et al., 2000).

## Methods

### Data Sources for MR Analyses and Selection of the Genetic Instruments

#### Leptin Levels

We performed the MR analyses based on the GWAS by Kilpeläinen et al. (Kilpeläinen et al., 2016), which included 32,161 individuals of European ancestry (stage 1). In stage 2, an additional 19,979 individuals with European ancestry were studied. The measured leptin level (µg/ml) was transformed logarithmically and adjusted for age, sex and study-specific covariates (e.g. genotype-derived principal components). Five loci were genome-wide associated (*p* < 5 × 10^−8^) with leptin levels: *LEP*, *SLC32A1*, *GCKR*, *CCNL1* and *FTO*. After adjustment of leptin levels for BMI, *FTO* did not remain significant. For genome-wide significant single nucleotide polymorphisms (SNP), results (non-adjusted and adjusted for BMI) were published for the total sample and separately for males and females. However, complete summary statistics for leptin levels with/without adjustment for BMI were only available for sex-combined datasets. SNP heritability (h^2^_SNP_) was not reported.

The most recent study on genetic determination of leptin levels was an exome-based analysis (EWAS) in individuals of diverse ancestries (Yaghootkar et al., 2020). This analysis was based on a total of 57,232 adults from 35 cohorts, of whom 50,321 were of European, 4,387 of African, 2,036 of East Asian, and 488 of Hispanic descent. In order to use the most recent currently available data, we performed MR using the SNPs from this study as an instrumental variable (IV). The studies included in the EWAS study computed residuals for leptin concentrations (in ng/mL) using linear regression, adjusting for age, genome-wide principal components, and any study specific covariates (e.g. study center). The residuals were calculated with and without adjustments for BMI. The residuals were transformed using rank based inverse normal transformation to follow a distribution with a mean of 0 and a standard deviation of 1. Results were reported for all ancestries combined and separately for Europeans and for both sexes and separately for women and men. In addition, three different models (additive, recessive and dominant) were calculated. An array-wide Bonferroni-corrected threshold of *p* < 2 × 10^−7^ for ∼250,000 variants was defined as significance level. Ten loci associated with leptin concentrations were found in the total sample, four of which had previously been detected by Kilpeläinen et al. (2016). Only nine variants were detected in Europeans [SNP rs17151919 (*LEP*) was not observed in Europeans], five and six of which attained significance after study-wide correction based on leptin levels unadjusted and adjusted for BMI, respectively. We used the effect sizes for unadjusted leptin levels determined in Europeans calculated with an additive model for our MR. How much of the total variance is explained by the SNPs (h^2^_SNP_) was not reported (Yaghootkar et al., 2020).

We used the results of GWAS and EWAS on leptin levels adjusted for BMI to assess the effect of BMI on leptin levels. First, we calculated MR with all SNPs that associated significantly with unadjusted leptin levels. In the second step, we considered only those SNPs that showed a significant effect on leptin levels even after adjustment for BMI (*p* < 0.05). As there is no GWAS for BMI-adjusted AN, we continued to use effect sizes for non-adjusted leptin levels here.

In addition, we performed MR with leptin levels in females only as exposure.

#### Sample Overlap (GWAS/EWAS)

According to the Supplementary tables in Kilpeläinen et al. (2016), a total of 28 studies were included in Stage 1 and Stage 2. Of these studies, 11 studies overlap with the EWAS by Yaghootkar et al. (2020). Considering the number of samples in each study, the sample overlap is 39%. Conversely, in EWAS, the 11 overlapping studies result in a sample overlap of 28%.

#### Anorexia Nervosa (AN)

We used the latest GWAS for AN (Watson et al., 2019) for both directions of MR analyses, i.e. using the complete summary statistics for the analysis considering AN as outcome variable and for the reverse MR analysis with AN as exposure considering the eight genome-wide significant SNPs for the definition of the genetic instrument. This GWAS included 16,992 patients with AN and 55,525 controls with European ancestry. Case definitions included a lifetime diagnosis of AN based on hospital or registry records, structured clinical interviews or online questionnaires based on standardized criteria (DSM-III-R, DSM-IV, ICD- 8, ICD-9 or ICD-10). In the UK Biobank, cases had self-reported a diagnosis of AN. Controls were matched to ancestry. In some control cohorts, individuals were screened for lifetime eating disorders and/or some or all psychiatric disorders. As AN is infrequent, large, unscreened control cohorts were considered suitable. About 88% of the cases are women, whereas the proportion of women and men in the controls was more balanced. Reported SNP heritability was 11–17% (Watson et al., 2019).

In case of unavailability of SNPs in GWAS for the outcome phenotype, we used proxy-SNPs (Hartwig et al., 2016). The SNPs with linkage disequilibrium (LD) of at least *r* ^2^ ≥ 0.80 (on the basis of GRCh37. p13, Ensemble version 87, 1,000 genomes: phase 3 version 5 for European ancestry) were extracted from the *in silico* tool SNIPA (Arnold et al., 2015) (http://www.snipa.org. Accessed between December 2020 and April 2021). Selection criteria for proxy-SNPs were defined: first highest *r* ^2^, second smallest distance to the lead SNP, third no strand-ambiguous or palindromic alleles (Hartwig et al., 2016).

#### Sample Overlap (Leptin Levels/AN)

There does not appear to be any sample overlap between the GWAS or EWAS on leptin levels and GWAS on AN (Kilpeläinen et al., 2016; Watson et al., 2019; Yaghootkar et al., 2020).

### Testing MR Assumptions and Statistical Analysis

To perform an MR study, three main assumptions have to be met (Lawlor et al., 2008; König and Greco, 2018): 1) The genetic instrument must have a true association with exposure. 2) The genetic instrument is independent of potential confounding factors in the relationship between the exposure and the outcome. 3) the outcome is associated with the genetic instrument only through the effect of the exposure (König and Greco, 2018). Therefore, the effect of the genetic instrument on the outcome has to be mediated only by the exposure and there should be no direct effects (Haycock et al., 2016).

Only the first assumption can be directly tested (Haycock et al., 2016). To fulfill this assumption, it is recommended to use independent genome-wide associated SNPs as instrumental variable (IV) for exposure (*p* < 5 × 10^−8^). We deviated from this threshold in part because some variants used as instruments did not achieve genome wide significance in some subgroups (e.g. Europeans or women) in contrast to the significant findings for the overall study group. Among these SNPs, we selected those with *p* < 0.05 and calculated the F-statistics in the next step for each of these SNPs using formula F = (beta/se)^2^. We excluded the SNPs with F < 10 from the MR (Swerdlow et al., 2016; Burgess et al., 2017b). Several approaches were applied to investigate assumptions two and three. We analysed horizontal pleiotropy by estimating the intercept of Egger’s regression. If Eggers intercept is not significantly different from zero, it can be assumed that there is no horizontal pleiotropy (Burgess and Thompson, 2017). In order to detect potential pleiotropic instruments, we also conducted MR PRESSO, which applies a global distortion test, to evaluate whether the removal of the potentially pleiotropic instrument leads to a significant difference in the overall causal estimate. Simulation studies showed that MR PRESSO is more sensitive to horizontal pleiotropy than MR Eggers intercept (Verbanck et al., 2018).

Genetic polymorphisms are sometimes associated with multiple aspects or dimensions of a single trait (Haycock et al., 2016). To test such heterogeneity of the IV, we used Cochran’s Q-statistic (Burgess et al., 2017a). This test examines whether causal estimates of genetic variants (SNPs) are comparative (Bowden et al., 2015).

It is highly implausible that all genetic variants used as IV in MR fulfill the instrumental variable assumptions. The different methods vary in their robustness to violations of the assumptions for MR. There is no method that can provide an infallible test of causality. Therefore, it is recommended to perform different methods to assess whether a causal effect as determined *via* MR is appropriate (Burgess et al., 2017a; Burgess et al., 2019). We carried out the following statistical methods: 1) Inverse-Variance Weighted method (IVW) assumes that all ratio estimates provide independent evidence on the causal effect and there is no pleiotropic effect. IVW thus assumes, that all genetic variants are valid instruments. There is no intercept term in the regression model (Burgess and Thompson, 2017). 2) MR-Egger estimates the intercept as part of the analyses. The intercept term is interpreted as the average pleiotropic effect of a genetic variant included in the analyses. The pleiotropic effect is the effect of the genetic variant on the outcome that is not mediated *via* the exposure. A non-zero intercept from MR-Egger shows that there is directional pleiotropy, or that IV assumptions are violated, or both (Bowden et al., 2016; Burgess and Thompson, 2017). If the intercept term is exactly equal to zero, then the MR-Egger estimate will equal the IVW estimate. Alternatively, if the pleiotropic effects are independently distributed from the genetic associations with the risk factor (InSIDE assumption: INstrument Strength Independent of Direct Effect), then the MR-Egger estimate will be a consistent estimate of the causal effect as the sample size and number of genetic variants both increase (Burgess and Thompson, 2017; Hartwig et al., 2017). Comparison of IVW and MR-Egger estimates is helpful to interpret results of a MR analysis (Burgess and Thompson, 2017). 3) The mode-based estimate (MBE; simple mode, weighted mode) is robust to horizontal pleiotropy in a different manner to that of the IVW, MR-Egger or weighted median methods (Hartwig et al., 2017). MBE estimates the true causal effect consistently given the assumption that across all instruments, the most frequent value of bias through pleiotropy is zero [ZEro-modal pleiotropy assumption (ZEMPA) is met] (Hartwig et al., 2017). We calculated both simple as well as weighted MBE. Simple MBE is less precise than weighted MBE, but simple MBE is less prone to bias due to violations of the InSIDE assumption. Comparing both methods is perceived as being useful, but requires care caution, since the simple MBE may in some cases be imprecise (Hartwig et al., 2017). 4) Median based estimators are consistent even when up to 50% of the information comes from invalid instrumental variables. Weighted median estimator has an efficiency similar to that of IVW method, simple median estimator is more inefficient compared with IVW and weighted median methods (Bowden et al., 2016); penalised weighted median estimator is more robust in the case of heterogeneity of IV(Rees et al., 2019). 5) Robust Adjusted Profile Score (MR RAPS) provides an overall estimator, which is robust against systematic and idiosyncratic pleiotropy (some genetic instruments have a large effect on outcome) (Zhao et al., 2020). 6) MR PRESSO (see above) (Verbanck et al., 2018).

To investigate the relationship between study accuracy and effect size, we created a funnel plot (Haycock et al., 2016). Asymmetry in the funnel plot indicates that assumptions for MR are not met (Katikireddi et al., 2018). To assess whether a single SNP had a large effect on the regression coefficients, we performed a leave-one-out approach. To conduct this analysis, we ran the IVW regression by omitting each genetic variant in turn (Burgess and Thompson, 2017).

Forest and scatter plots were used to visualize combined results of single and multi-SNP analyses. The scatter plots show the single SNP effects on the exposure against the single SNP effects on the outcome with corresponding standard deviations and estimated regression lines of the multi-SNP analyses.

The power analysis estimated whether the analysis, given sample size, proportion of cases in the study, and the proportion of variance explained, provides sufficient statistical power to detect a true casual effect (Brion et al., 2013). Because the proportion of variance explained (h^2^_SNP_) was not published in the papers on GWAS and EWAS on leptin levels, we calculated these using the LDSC tool (Bulik-Sullivan et al., 2015).

In reporting this analysis, we followed the recommendations of STROBE-MR: Guidelines for strengthening the reporting of Mendelian randomization studies for two-sample MR (Davey Smith et al., 2019). The tests were performed using the software “R”, version 3.5.2, and R-package “TwoSampleMR” (https://github.com/MRCIEU/TwoSampleMR) (Hemani et al., 2018). MR PRESSO was performed with R-package “rondolab/MR-PRESSO” (https://github.com/rondolab/MR-PRESSO) (Verbanck et al., 2018). The h2SNP was calculated with LDSC tool (https://github.com/bulik/ldsc) (Bulik-Sullivan et al., 2015).

## Results

**FIGURE 1:**
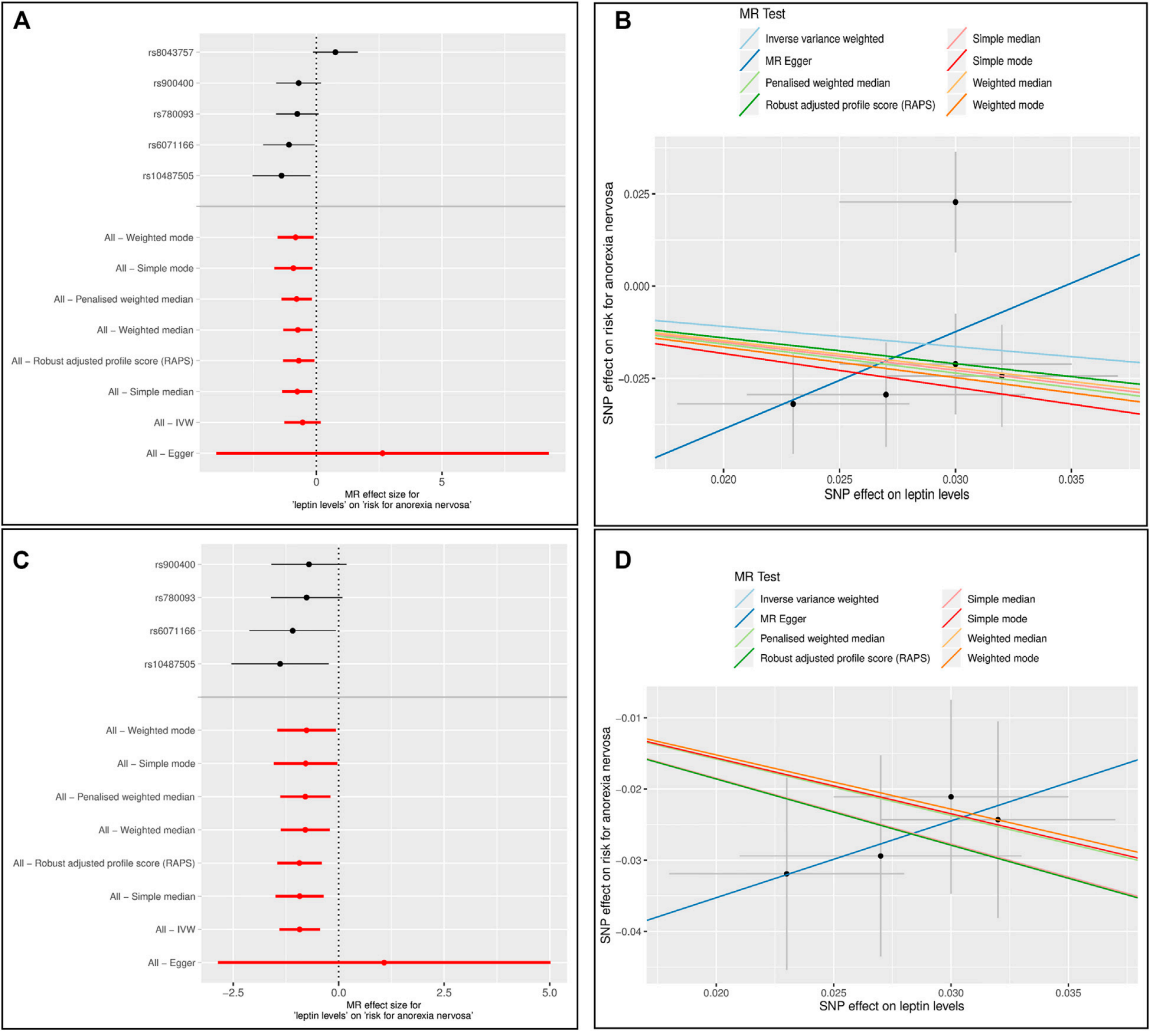
Results of the single and multiple-SNP Mendelian randomization (MR) analyses for effect of leptin levels (Kilpeläinen et al., 2016) on risk for anorexia nervosa (AN) (Watson et al., 2019). The black lines in forest plots **(left side)** visualize the results of single SNP analyses; the red lines visualize the results of the multiple SNP analysis. In scatter plots **(right side)** the slopes of each line represent the causal association for different MR method **(A)** and **(B)**: Instrument Variable (IV): 5 SNPs associated with unadjusted leptin levels **(C)** and **(D)**: Instrument Variable (IV): 4 SNPs associated with unadjusted leptin levels. The SNP rs8043757 (*FTO*) was excluded.

In the analysis with leptin level as exposure (Kilpeläinen et al., 2016) and risk for AN as outcome including five SNPs (Table 1), the results were inconsistent depending on the method of analysis (Table 2). Simple median, weighted median, penalized weighted median, MR PRESSO and MR RAPS revealed a significant causal effect of low leptin levels on the risk of AN; however, MR Egger, Inverse variance weighted (IVW), simple mode and weighted mode did not (Table 2; Figures 1A,B). Single SNP analyses showed that two SNPs were significant: rs10487505 (*LEP*) and rs6071166 (*SLC32A1*). The MR Eggers intercept showed no evidence of pleiotropy, but MR PRESSO identified rs8043757 (*FTO*) as an outlier and calculated outlier-corrected MR estimates: *p* = 0.008. The heterogeneity tests were significant. The overall effect calculated with the penalised weighted median method, which is robust for heterogeneity, was significant (Table 2). Since the SNP near *LEP* rs10487505:C:G is palindromic, we inspected the effect directions for SNPs in high LD (rs791600:G:A and rs2167289:G:T; *r* ^2^ = 0.98); the effect directions for leptin and AN were the same as for rs10487505. Funnel and leave-one-out plots are shown in Supplementary Figures 1,2. The h^2^_SNP_ calculated with the LDSC tool for GWAS on leptin levels was 0.097 (genomic inflation factor *λ* = 1.0574). Our analysis had a power of 80% to detect an OR of 1.08 for AN per 1 standard deviation decrease in leptin levels and 100% power to detect an OR of 1.14 (Supplementary Figure 3).

**TABLE 1:** Single nucleotide polymorphisms (SNPs) associated with leptin levels [unadjusted and adjusted for BMI, µg/ml (log-transformation)] (Kilpeläinen et al., 2016) and their association with the risk for anorexia nervosa (AN) (OR is transformed to beta) (Watson et al., 2019).

SNP	Gene	Chr	EA	OA	Leptin levels unadjusted for BMI					Leptin levels adjusted for BMI			AN		
					EAF	B	se	*p*	F	b	se	*p*	b	se	*p*
rs10487505	*LEP*	7	G	C	0.50	0.023	0.005	9.0E-06	21.16	*0.029*	*0.004*	*2.0E-12*	−0.032	0.0135	0.0184
rs6071166	*SLC32A1*	20	C	A	0.37	0.027	0.006	6.6E-07	20.25	*0.024*	*0.004*	*1.8E-08*	−0.029	0.0141	0.0378
rs780093	*GCKR*	2	C	T	0.61	0.032	0.005	2.3E-10	40.96	*0.024*	*0.004*	*3.8E-10*	−0.024	0.0138	0.0778
rs8043757	*FTO*	16	T	A	0.40	0.030	0.005	1.1E-09	36.00	*0.001*	*0.004*	*8.4E-01*	0.023	0.0136	0.0925
rs900400	*CCNL1*	3	T	C	0.60	0.030	0.005	5.6E-09	36.00	*0.021*	*0.004*	*1.2E-07*	−0.021	0.0136	0.1204

Font in italic: these effect sizes were used to assess the effect of BMI on leptin levels; Chr: chromosome; EA: effect allele; OA: other allele; b: effect size of EA; se: standard error; p: p-value; F: F-statistics.

**TABLE 2:** Results of single SNP MR analyses and the overall causal effect of leptin levels (Kilpeläinen et al., 2016) on the risk for anorexia nervosa (AN) (Watson et al., 2019) calculated using different MR methods. The beta estimates the change in risk for AN (OR is transformed to beta) per change of 1 unit of leptin concentration (log-transformed µg/ml).

SNP and nearest gene		Effect of leptin levels on risk for AN (SNP n = 5)					Effect of leptin levels on risk for AN (the SNP rs8043757 was excluded)				
		b	Se	*p*	Lower 95% CI	Upper 95% CI	b	se	*p*	Lower 95% CI	Upper 95% CI
**rs10487505**	*LEP*	−1.387	0.587	**0.018**			−1.387	0.587	**0.018**		
**rs6071166**	*SLC32A1*	−1.089	0.522	**0.037**			−1.089	0.522	**0.037**		
rs780093	*GCKR*	−0.759	0.431	0.078			−0.759	0.431	0.078		
rs900400	*CCNL1*	−0.703	0.453	0.121			−0.703	0.453	0.121		
rs8043757	*FTO*	0.760	0.453	0.094							
MR Egger		2.635	3.375	0.492	−3.981	9.251	1.078	2.009	0.645	−2.859	5.016
Inverse variance weighted (IVW)		−0.546	0.369	0.139	−1.269	0.177	−0.923	0.244	**1.5E-04**	−1.401	−0.445
Simple median		−0.759	0.318	**0.017**	−1.383	−0.136	−0.924	0.328	**0.005**	−1.566	−0.282
Weighted median		−0.737	0.280	**0.009**	−1.287	−0.188	−0.790	0.298	**0.008**	−1.375	−0.206
Penalised weighted median		−0.785	0.307	**0.011**	−1.387	−0.183	−0.790	0.308	**0.010**	−1.395	−0.186
Simple mode		−0.913	0.352	0.060	−1.603	−0.223	−0.782	0.383	0.134	−1.533	−0.032
Weighted mode		−0.826	0.373	0.091	−1.556	−0.096	−0.761	0.373	0.134	−1.492	−0.029
MR RAPS		−0.700	0.319	**0.028**	−1.326	−0.074	−0.930	0.268	**0.001**	−1.455	−0.404
MR PRESSO outlier corrected		−0.923	SD = 0.148	**0.008**							
		MR PRESSO global test *p* = 0.023; Eggers intercept = −0.091, SE = 0.096 *p* = 0.413; Heterogeneity test based on MR Egger: Q (df = 3) = 9.078 (df = 5), *p* = 0.028; based on IVW Q (df = 4) = 11.798, *p* = 0.019					MR PRESSO no outlier; Eggers intercept = −0.057, SE = 0.057, *p* = 0.421; Heterogeneity test based on MR Egger Q (df = 2) = 0.097, *p* = 0.953); based on IVW (Q (df = 3) = 1.105, *p* = 0.776				

b: effect size; se: standard error; *p*: *p*-value; CI: confidence interval. Bolded values: *p* < 0.05.

The GWAS on BMI-adjusted leptin levels showed that rs8043757 was no longer significantly associated with leptin levels (Table 1). MR PRESSO had also identified this SNP as pleiotropic. Thus, rs8043757 does not fulfill the assumptions for IV, so we repeated the MR without this SNP. A causal effect was found with five MR methods (IVW, simple median, weighted median, penalised weighted median and RAPS) (Table 2; Figures 1C,D) Eggers intercept was not significant, also MR PRESSO detected no outliers. Heterogeneity test was not significant. The funnel and leave-one-out plots are shown in Supplementary Figures 4,5.

**FIGURE 2:**
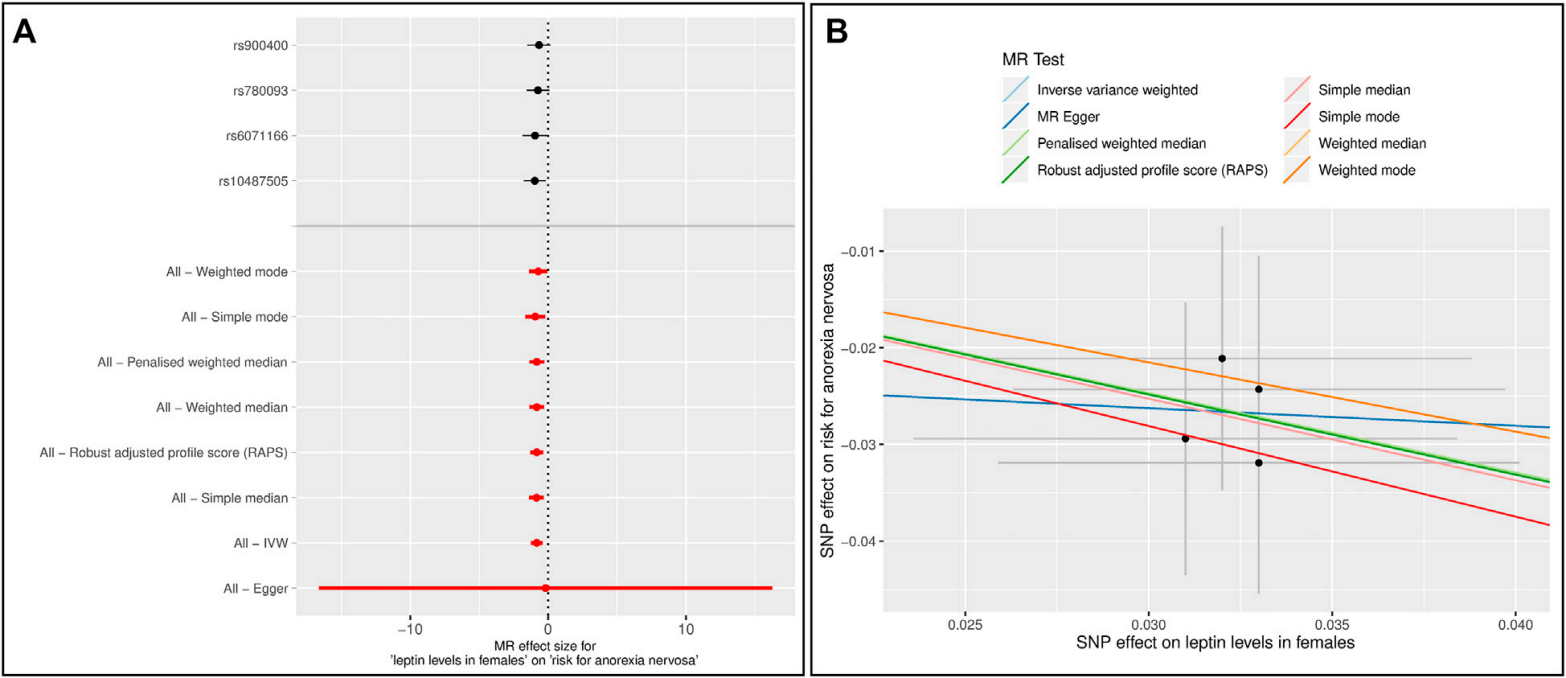
Results of the single and multiple-SNP Mendelian randomization (MR) analyses for effect of leptin levels in females (Kilpeläinen et al., 2016) on risk for anorexia nervosa (AN) (Watson et al., 2019). The SNP rs8043757 (*FTO*) was excluded **(A)** The black lines in forest plot visualize the results of single SNP analyses; the red lines visualize the results of the multiple SNP analysis **(B)** In scatter plot the slopes of each line represent the causal association for different MR method.

Since most of the participants in the GWAS for AN are female, we performed MR analyses with data on leptin levels retrieved only from women (Supplementary Table 1) (Kilpeläinen et al., 2016). Six MR methods (IVW, all median based methods, simple mode and RAPS) showed a significant effect of leptin levels on the risk for AN (Supplementary Table 2, Supplementary Figures 6–9). In the single SNP analysis, rs10487505 and rs6071166 were again significant. After excluding the SNP rs8043757, the results were similar, except that the *p*-value from the simple mode method was no longer significant. However, the overall effect sizes were slightly higher (Figure 2, Supplementary Table 3, Supplementary Figures 10,11). In both analyses, there was no evidence of pleiotropy and heterogeneity.

We also used the SNPs from the exome study by (Yaghootkar et al., 2020) as genetic instruments (Table 3). Loci *LEP*, *GCKR*, *CCNL1* and *FTO* were detected by both Kilpeläinen et al. (2016) and Yaghootkar et al. (2020), albeit with different SNPs. The loci *KLHL31* (lead SNP rs3799260), *ZNF800* (lead SNP rs62621812), *ACTL9* (lead SNP rs234055) and *KLF14* (lead SNP rs972283) had not been detected by (Kilpeläinen et al., 2016) and revealed novel genetic associations with leptin levels. However, for Europeans, SNPs rs3799260 (*KLHL31*) and rs234055 (*ACTL9*) do not seem to be particularly relevant, at least they appear to be weak IV (F < 10).

**TABLE 3:** Single nucleotide polymorphisms (SNPs) associated with leptin levels [unadjusted and adjusted for BMI, ng/ml (rank based inverse normal transformation)] derived by the EWAS (Yaghootkar et al., 2020) and their association with the risk for anorexia nervosa (AN) (OR is transformed to beta) (Watson et al., 2019).

SNP^a^	Chr	Nearest gene	EA	OA	EAF	Leptin levels unadjusted for BMI^b^				Leptin levels adjusted for BMI^b^			Anorexia nervosa		
						B	se	*p*	F^c^	b	se	*p*	b	se	*p*
rs1121980	16	*FTO*	A	G	0.432	0.055	0.007	7.7E-17	61.73	*0.005*	*0.007*	*4.5E-01*	0.016	0.0135	0.2379
rs1260326	2	*GCKR*	C	T	0.607	0.032	0.007	1.7E-06	20.90	*0.048*	*0.007*	*4.3E-13*	−0.022	0.0138	0.1057
rs13389219	2	*COBLL1*	T	C	0.394	0.046	0.007	7.3E-11	43.18	*0.053*	*0.007*	*1.1E-13*	0.004	0.0136	0.7920
rs2340550	19	*ACTL9*	G	A	0.685	−0.008	0.007	2.8E-01	1.31	−*0.016*	*0.007*	*2.6E-02*	−0.009	0.0143	0.5494
rs3799260	6	*KLHL31*	T	C	0.818	−0.024	0.008	3.8E-03	9.00	−*0.038*	*0.008*	*3.8E-06*	0.004	0.0171	0.8049
rs62621812	7	*ZNF800*	A	G	0.031	−0.098	0.018	8.2E-08	29.64	−*0.127*	*0.018*	*2.8E-12*	−0.008	0.0435	0.8495
rs791600	7	*LEP*	A	G	0.411	−0.043	0.007	1.4E-09	37.73	−*0.063*	*0.007*	*5.4E-19*	0.039	0.0136	0.0043
rs900399	3	*CCNL1*	G	A	0.396	−0.033	0.007	2.4E-06	22.22	−*0.040*	*0.007*	*9.2E-09*	0.021	0.0136	0.1184
rs972283	7	*KLF14*	G	A	0.521	−0.041	0.006	1.1E-10	46.69	−*0.056*	*0.006*	*3.8E-18*	−0.009	0.0135	0.5017

Font in italic: these effect sizes were used to assess the effect of BMI on leptin levels; Chr: chromosome; EA: Effect allele; OA: other allele; b: effect size of EA; se: standard error; *p*: *p*-value; F: F-statistics.

a These SNPs were significant in at least one of the sub-analyses by Yaghootkar et al. (2020).

b Effect sizes have been calculated for subjects with European ancestry with the additive model.

c Only SNPs with F > 10 were included as IV in MR.

MR analysis did not detect an overall effect of leptin levels on the risk for AN. In the single SNP analysis, SNP rs791600 (*LEP*) was significantly associated with AN (beta = -0.905, se = 0.316, *p* = 0.004) (Table 4, Supplementary Figures 12–15). There is no evidence of pleiotropy. Heterogeneity test based on IVW was significant (*p* = 0.029). The h^2^_SNP_ for EWAS on leptin levels calculated with the LDSC tool was 0.1913 (genomic inflation factor *λ* = 1.1612). Power analysis showed that our MR with EWAS data had a power of 80% to detect an OR of 1.057 and a power of 100% to detect an OR of 1.10 for AN per 1 standard deviation decrease in leptin level (Supplementary Figure 16).

**TABLE 4:** Results of single SNP MR analyses and the overall causal effect of leptin levels (EWAS) (Yaghootkar et al., 2020) on the risk for anorexia nervosa (AN) (Watson et al., 2019) calculated using different MR methods. The beta estimates the change in risk for AN (OR is transformed to beta) per change of 1 SD in leptin level [ng/ml (rank based inverse normal transformation)].

SNP and nearest gene		Effect of leptin levels on risk for AN (SNP n = 7)					Effect of leptin levels on risk for AN (the SNP rs1121980 was excluded)				
		B	se	*p*	Lower 95% CI	Upper 95% CI	b	Se	*p*	Lower 95% CI	Upper 95% CI
rs1260326	*GCKR*	−0.697	0.431	0.106			−0.697	0.431	0.106		
rs13389219	*COBLL1*	0.078	0.296	0.791			0.078	0.296	0.791		
rs62621812	*ZNF800*	0.085	0.444	0.849			0.085	0.444	0.849		
**rs791600**	***LEP***	−**0.905**	**0.316**	**0.004**			−**0.905**	**0.316**	**0.004**		
rs900399	*CCNL1*	−0.643	0.412	0.119			−0.643	0.412	0.119		
rs972283	*KLF14*	0.222	0.329	0.500			0.222	0.329	0.500		
rs1121980	*FTO*	0.289	0.245	0.239							
MR Egger		0.986	0.713	0.225	−0.412	2.385	0.607	0.866	0.522	−1.090	2.305
Inverse variance weighted		−0.132	0.192	0.491	−0.508	0.244	−0.281	0.207	0.175	−0.687	0.125
Simple median		0.078	0.223	0.726	−0.359	0.515	−0.282	0.208	0.175	−0.690	0.126
Weighted median		0.083	0.178	0.641	−0.265	0.431	−0.098	0.218	0.652	−0.526	0.329
Penalised weighted median		0.115	0.177	0.517	−0.232	0.461	0.078	0.215	0.715	−0.343	0.500
Simple mode		0.166	0.268	0.560	−0.360	0.692	0.099	0.444	0.833	−0.772	0.969
Weighted mode		0.193	0.187	0.342	−0.174	0.560	0.116	0.292	0.707	−0.456	0.688
MR RAPS		−0.099	0.201	0.623	−0.493	0.295	−0.250	0.221	0.258	−0.682	0.183
		MR PRESSO no outlier Eggers intercept = −0.051, SE = 0.031, *p* = 0.167 Heterogeneity test based on MR Egger Q (df = 5) = 9.241, *p* = 0.100; IVW: Q (df = 6) = 14.058, *p* = 0.029					MR PRESSO no outlier Eggers intercept = 0.038, SE = 0.036, *p* = 0.351 Heterogeneity test based on MR Egger Q (df = 4) = 7.879, *p* = 0.096, based on IVW Q (df = 5) = 10.074, *p* = 0.073				

b: effect size; se: standard error; *p*: *p*-value; CI: confidence interval b: effect size; se: standard error; *p*: *p*-value; CI: confidence interval. Bolded values: *p* < 0.05.

**FIGURE 3:**
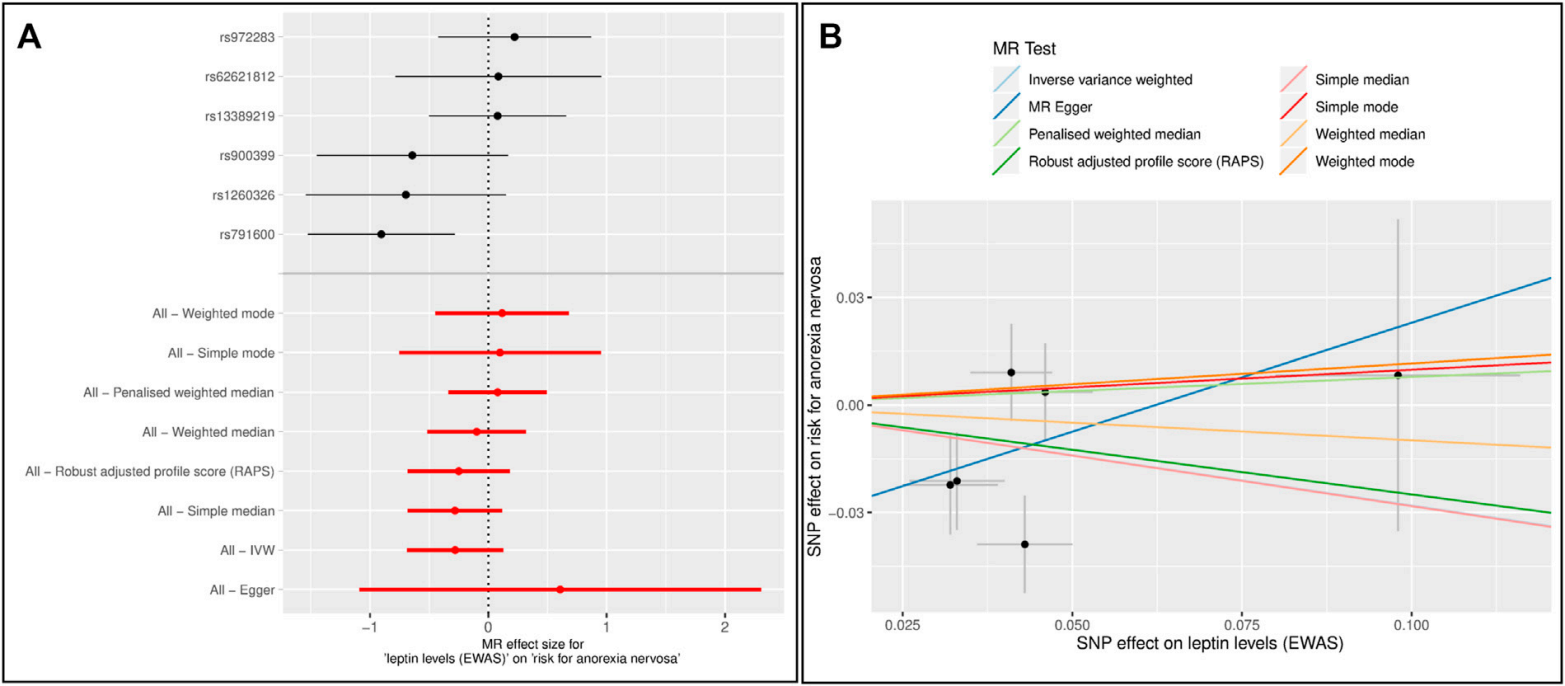
Results of single and multiple-SNP Mendelian randomization (MR) analyses for effect of leptin levels (Yaghootkar et al., 2020) on risk for anorexia nervosa (AN) (Watson et al., 2019). The SNP rs1121980 (*FTO*) was excluded **(A)** The black lines in forest plot visualize the results of single SNP analyses; the red lines visualize the results of the multiple SNP analysis **(B)** In scatter plot the slopes of each line represent the causal association for different MR method.

Because the SNP rs1121980 (*FTO*) was no longer significant in EWAS for leptin levels adjusted for BMI, we repeated the MR without this SNP (Table 3). Again, there was no overall effect of leptin on risk for AN (Table 4, Figures 3, Supplementary Figures 17,18). Evidence of horizontal pleiotropy or heterogeneity was not found.

The analyses using effect sizes for SNPs associated with leptin levels identified in the EWAS were also performed in females only (Supplementary Table 4). Again, no significant finding emerged. The SNP rs791600 (*LEP*) was again significant in the single SNP analysis in both analyses based on 1) all SNPs or 2) without rs1121980 (*FTO*) (Supplementary Tables 5–7, Supplementary Figures 19–22; Supplementary Figures 23–26).

**FIGURE 4:**
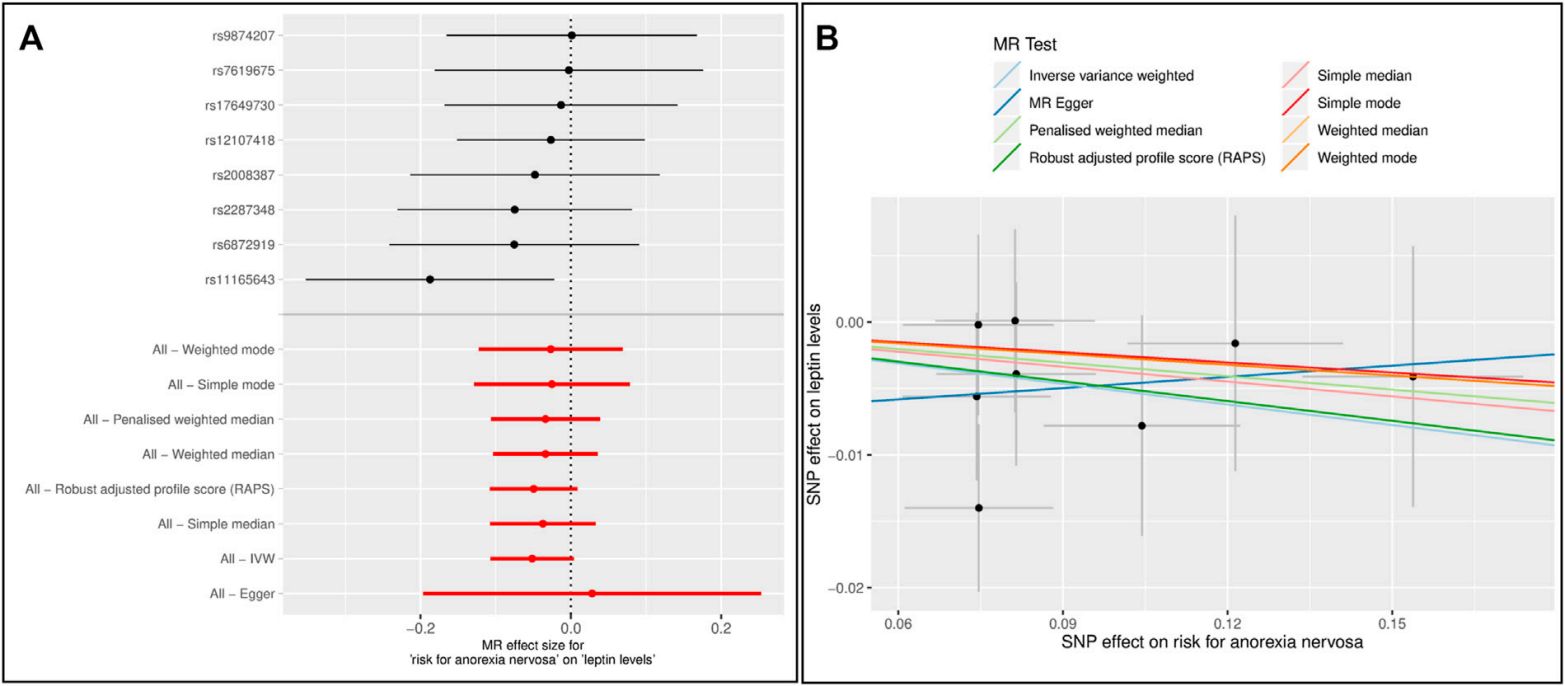
Results of the single and multiple-SNP Mendelian randomization (MR) analyses for the SNP effect of anorexia nervosa (AN) (Watson et al., 2019) on leptin levels (Kilpeläinen et al., 2016) **(A)** The black lines in forest plot visualize the results of single SNP analyses; the red lines visualize the results of the multiple SNP analysis **(B)** In scatter plot the slopes of each line represent the causal association for different MR method.

In the reverse MR analyses, there was no causal effect of a higher risk of AN on leptin levels using the GWAS data (Figures 4, Supplementary Tables 8,9, Supplementary Figures 27,28). There was no evidence of either horizontal pleiotropy or heterogeneity of genetic instruments.

The reverse analysis of the effect of risk of AN on leptin levels using EWAS could not be performed because the SNPs associated with AN (Watson et al., 2019) are not included in EWAS (Yaghootkar et al., 2020). We could only find proxies (LD ≥ 0.8) for two SNPs (rs10747478 and rs9821797), which would not have been sufficient for MR analyses.

### Sensitivity Analyses

We performed two sensitivity analyses where we used the loci *LEP*, *GCKR* and *CCNL1* which were detected in both GWAS and EWAS as IV. We did not include the SNPs rs8043757 and rs1121980 (*FTO*). In the first MR we used SNPs rs10487505 (*LEP*), rs780093 (*GCKR*), rs900400 (*CCNL1*) detected in GWAS by Kilpeläinen et al. (2016). In the second MR SNPs rs791600 (*LEP*), rs1260326 (*GCKR*) and rs900399 (*CCNL1*) reported by Yaghootkar et al. (2020) were included. These two analyses yielded similar results: MR Egger and mode-based estimators were not significant; other overall MR estimators showed that low leptin level increased the risk for AN (Supplementary Tables 10,11; Supplementary Figures 29–36).

For further sensitivity analyses we proceeded as follows: as IV we used SNPs that were reported as significant in EWAS (Yaghootkar et al., 2020), but for MR we used the effect sizes from GWAS (Kilpeläinen et al., 2016) (Supplementary Table 12). In this MR analysis, only median-based methods showed a significant effect of leptin levels on the risk of AN (Supplementary Table 13, Supplementary Figures 37–40). Other MR methods were not significant. The reverse analysis (SNPs from GWAS on leptin levels with effect sizes from EWAS as IV for exposure on risk for AN) was not possible because only two SNPs (rs780093, rs900400) from GWAS were also reported in the EWAS. In the EWAS there were also no proxies for the other SNPs.

## Discussion

Our MR analyses suggest a causal effect of lower leptin levels for risk of AN using data from GWAS (Kilpeläinen et al., 2016; Watson et al., 2019). This also applied upon restriction to leptin SNP data based on females only. We performed our MR analysis with different MR methods to take into account respective strengths and weaknesses of the statistical methods against violation of the assumptions for MR (Burgess et al., 2017a). We also carried out different sensitivity analyses to take into account the specificities of the datasets. Not every MR method showed a significant effect. This is due to properties of the methods with respect to the characteristics of the data used.

In the analysis with leptin levels unadjusted for BMI as exposure and AN as outcome, IV heterogeneity and horizontal pleiotropy seemed to be an issue. The outlier-corrected overall estimator [MR-PRESSO (Verbanck et al., 2018)] was significant after excluding pleiotropic SNP rs8043757 (*FTO*). MR-RAPS, whose estimator is also more robust to pleiotropy (Zhao et al., 2019), showed a significant causal effect of leptin levels on the risk for AN. A significant effect was also observed upon use of the weighted median methods, of which the penalized weighted median method is more robust to heterogeneity of IV (Rees et al., 2019). These methods show that the weighted majority of genetic variants are associated with the outcome (Burgess and Thompson, 2017). Since pleiotropy is obviously an issue in this analysis, the assumptions for IVW were not met. The overall estimator from MR Egger was not significant, but the direction was positive, unlike the estimators from the analyses with other methods. This confirms the suspicion of the violation of the assumptions for IV, because an influential genetic variant changes the sign of the MR-Egger estimate (Burgess and Thompson, 2017). Upon consideration of all MR results giving the same direction of effect (except for MR-Egger), we interpret the findings using data from GWAS as indicative of an elevated risk for development of AN due to factors associated with low leptin levels.

After excluding pleiotropic SNP rs8043757 (*FTO*), the difficulties with horizontal pleiotropy and heterogeneity were no longer evident. The estimators of MBE and MR Egger were not significant, however IVW, RAPS, all median-based methods (single, weighted and penalised weighted median) showed that there is a significant causal effect of lower leptin levels on higher risk for AN. The analyses based on females only showed similar results [mode-based methods and MR Egger were not significant, IVW, RAPS, all median-based methods (single, weighted and penalised weighted median) were significant]. We again conclude that our results indicate that lower leptin levels may have a causal effect on higher risk of AN, and this is also independent of BMI.

We were unable to assess the effects of leptin levels on the risk of AN in women compared to men due to lack of data. Such a study would be possible with availability of sex-dependent analyses of AN-GWAS. Such a GWAS including only affected females (or males) and sex-matched healthy controls could help to clarify whether the substantially lower risk for AN in men can also in part be explained by differences in genetic factors. It should be mentioned that in GWAS on AN (Watson et al., 2019) 12% of the cases were men. The GWAS on leptin levels (Kilpeläinen et al., 2016) revealed that only SNP rs10487505 (*LEP*) resulted in different effect sizes and *p*-values in women and men (Kilpeläinen et al., 2016). Yaghootkar et al. (2020) reported two genes (*CNTD1*, *DNAJC18*), which may show sex-differences in associations with leptin levels. Upon use of the GWAS (Kilpeläinen et al., 2016), our MR study was able to show the effect of leptin levels based on females only on the risk for AN, despite lower power.

Single SNP analyses showed that rs10487505 and rs6071166 were consistently associated with risk for AN in all analyses. The common variant rs10487505 (7:128220110) is located 21 kb from *LEP* (Kilpeläinen et al., 2016) within a regulatory region. The functional implications of the association of SNP rs10487505 in *LEP* are not yet understood. MicroRNA 129-1 (7:128207872-128207943) is located upstream to rs10487505. This microRNA has been associated with e.g. glycerol levels (Raitoharju et al., 2014), type II diabetes mellitus (Hara et al., 2014), educational attainment and cognitive function (Lee et al., 2018) and tumorigenesis of retinoblastoma (Zhao et al., 2009). More likely than microRNA 129-1, the interplay of long noncoding RNA (IncOb), enhancer sequences (LE1 and LE2) and LEP promoter transcript, which are also located in this region, could be relevant for the expression of LEP (Dallner et al., 2019). Thus, higher expression of the IncOb was associated with higher leptin mRNA levels in fed, fasted, and obese mice (Dallner et al., 2019). The IncOb is located upstream (−28 kb) of the LE1, which in turn is upstream from *LEP*. The enhancer LE2 is located downstream from *LEP*. Both enhancers interact with the proximal leptin promoter, which is required for proper leptin expression (de la Brousse et al., 1996; Mason et al., 1998; Munzberg and Heymsfield, 2019). The proximal promoter alone is not sufficient for adipose-specific leptin expression: at least one enhancer (LE1 or LE2) is required to maintain leptin expression in adipose tissue. IncOb binds exclusively to the promoter. The absence of IncOb significantly decreases leptin gene expression, therefore IncOb seems to be required to exploit the full capacity of leptin expression (Dallner et al., 2019; Munzberg and Heymsfield, 2019). Expression of the analog human lncRNA (EST EL947753) is correlated with leptin mRNA expression in human adipose tissue. The variant rs10487505, which showed the lowest *p*-value and largest effect size with BMI-adjusted leptin levels, is located in this lncRNA.

The role of leptin levels for risk of AN was previously hypothesized in a GWAS on stringently phenotyped patients with AN that provided hints for a dysregulation of leptin signaling in this eating disorder (Li et al., 2017). However, the detected SNP (rs929626 in *EBF1*) did not reach genome-wide significance (*p* = 2.04 × 10^−7^) for AN.

The other consistently significant single SNP, SNP rs6071166 (20q11.23), is an intergenic variant ∼20 kb from *SLC32A1* (Kilpeläinen et al., 2016). However, the knockdown of the Adig gene [Adipogenin; alias *SMAF1* (Small Adipocyte Factor 1)] in mouse models decreased Lep expression and leptin secretion and thus provided evidence that *ADIG* controls leptin levels. The *ADIG* gene is located ∼116 kb from rs6071166 (Kilpeläinen et al., 2016) and plays a role in adipocyte differentiation and development (Kim et al., 2004).

We can only speculate why the analysis with genetic instruments from the EWAS did not show the overall effect of leptin levels on AN. The study by Yaghootkar et al. (2020) analysed the samples with the HumanExome BeadChip, which takes into account variants in gene-coding regions (some versions of the chip apparently also take into account SNPs outside of exons). This study also revealed that the *LEP* gene has a causal effect on the risk of AN: SNP rs791600, an intergenic variant (intron variant of predicted ncRNA LOC105375494) near the *LEP* gene, was significantly associated in the single SNP analyses. SNP rs791600 is in linkage disequilibrium (LD) (EUR *r* ^2^ = 0.70) with rs10487505 (see above). Since the EWAS contained SNPs from protein-coding regions and only a few selected intronic/intergenic SNPs, most intergenic SNPs are consequently missing from the study: both rs10487505 and rs6071166 (*SLC32A1*) were not included in the exome array. In addition to the *LEP* locus, the *GCKR* and *CCNL1* loci were identified in both the GWAS by Kilpeläinen et al. (2016) and in the EWAS by Yaghootkar et al. (2020), albeit the lead SNPs being different. The EWAS reported four novel loci in Europeans: rs62621812 (*p* = 8.2 × 10^−8^, after adjusting for BMI *p* = 2.8 × 10^−12^, *ZNF800*, missense), rs379926 (*p* = 3.8 × 10^−3^, after adjusting for BMI *p* = 3.8 × 10^−6^, *KLHL31*, missense), rs972283 (*p* = 1.1 × 10^−10^, after adjusting for BMI *p* = 3.8 × 10^−18^, *KLF14*, intergenic), and rs2340550 (only nominally significant after adjusting for BMI *p* = 2.6 × 10^−02^, *ACTL9*, missense; not included in MR). SNPs rs62621812 and rs972283 revealed non-significant positive associations between leptin level and risk of AN in single-SNP MR. In addition, as already mentioned, EWAS does not include the SNP rs6071166 (*SLC32A1*), which showed a significant negative effect on the risk of AN in single-SNP analyses. Sensitivity analyses for overlapping loci (*LEP*, *GCKR* and *CCNL1*) from GWAS and EWAS as IV support the suggestion that there is an effect of lower leptin levels on risk for AN. Further sensitivity analysis with SNPs from EWAS as IV, but with effect sizes from GWAS could not find a significant effect with most MR methods. Reverse analysis with SNPs from GWAS, but effect sizes from EWAS was not possible due to lack of data. More powerful GWAS are required to resolve the discrepancy between our results based on these two approaches and to figure out whether our MR results based on the GWAS by Kilpeläinen et al. (2016) are false positive.

For a SNP to be a valid IV, it does not necessarily have to be located in a protein-coding region. A SNP that is appropriate as an IV can also act as a *cis*, located close to the encoding gene influencing mRNA expression. However, *trans*-acting SNPs located outside the target gene or even on a different chromosome can also serve as suitable genetic instruments. However, these SNPs are more likely to be pleiotropic (Swerdlow et al., 2016). The more recent EWAS study can be advantageous as a data basis for IV, if they take less frequent variants into account (Swerdlow et al., 2016). For the GWAS by Kilpeläinen et al. (2016), different genotyping platforms included variants with at least MAF >1% or >5%, depending on the cohort. No information could be found on the different exome genotyping arrays used for the genotyping of the cohorts in EWAS (Yaghootkar et al., 2020) that would indicate that SNPs with MAF <1% were taken into account. Thus, there is no evidence regarding this aspect that MR analysis with SNPs from EWAS is by definition superior to MR analysis with genetic tools from GWAS.

With regard to the number of subjects, GWAS (Kilpeläinen et al., 2016) and EWAS (Yaghootkar et al., 2020) were similar in size. The power analysis showed that the IV from EWAS had slightly higher power to detect the true effect (EWAS: 100% power to detect OR = 1.10 vs GWAS: OR = 1.14). It should be pointed out that the genomic inflation rate for EWAS was elevated, which may lead to an overestimation of the h^2^_SNP_.

One reason we did not find an effect with EWAS data, if it exists, could be due to the rank-based inverse normal transformation of the leptin level measurements. Due to this transformation, the beta estimator cannot accurately reflect the effect per allele on the trait.

Our analyses (GWAS and EWAS) showed that the leptin level-reducing alleles of SNPs that are close to *LEP* associate with a higher risk of AN. The detection of a causal role for AN of variants in linkage disequilibrium with an effect on leptin levels in both GWAS and EWAS is in itself an important finding linking the two phenotypes. Based on our MR analyses, we conclude that low leptin levels may well increase the risk for AN. These results would further support the labeling of AN as a metabo-psychiatric disorder (Watson et al., 2019).

The causal reason for the observed hypoleptinemia in patients with acute AN (Hebebrand et al., 1997; Balligand et al., 1998; Misra and Klibanski, 2014) is not the disease AN per se, but the associated starvation, which entails a decreased body fat. However, based on the results of this study, a low endogenous leptin synthesis may represent a risk factor for the development of AN. Potentially young females [and potentially males, too, albeit with a much lower a priori risk (Nicholls et al., 2011; Steinhausen and Jensen, 2015)] with low leptin levels are at a higher risk for AN due to their genetically lower leptin levels (Kilpeläinen et al., 2016) and/or dysregulated leptin signaling (Li et al., 2017) in addition to other risk factors [e.g. personality traits, emotion dysregulation, hormonal and metabolic effects (Wonderlich et al., 2005; Thompson-Brenner et al., 2008; Miller, 2011; Lavender et al., 2015)]. For such individuals, self-induced weight loss (experimenting with diets, high physical activity/sport) may entail an increased risk of the onset of hypoleptinemia, which could be a relevant factor for patients to become entrapped in AN (Hebebrand et al., 2019). Physical activity has been shown to lead to lower levels of leptin (Lichtenstein et al., 2015), which may also increase the risk of AN in predisposed persons (Davis et al., 1994; Vayalapalli et al., 2018). As AN evolves, leptin levels drop into a critical range as a consequence of loss of body fat due to a negative energy balance. In the state of starvation associated with low body fat and hypoleptinemia, a positive energy balance only slowly entails increments in leptin levels upon re-alimantation (Hebebrand et al., 1997; Balligand et al., 1998), patients remain entrapped in the disease for a prolonged period of time. This slow increase of leptin secretion may underlie the slow improvement of patients during realimentation. The finding that an increase in leptin levels through exogenous application of recombinant human leptin *via* off-label treatment with human recombinant leptin (metreleptin) allows an escape from the “cage” (Milos et al., 2020; Antel et al., 2021) supports this hypothesis. In conclusion, a genetically lower leptin level in patients may not only represent a risk factor for a more rapid entrenchment in the eating disorder due to the in comparison to non-predisposed individuals earlier development of hypoleptinemia and as a consequence the initiation of central effects of starvation. The genetic predisposition to low leptin levels may also contribute to the slow recovery among realimentation. Finally, this genetic predisposition may play a role in the frequent relapses observed in AN.

## Limitations

When interpreting the results, it is important to note that the studies included in GWAS on leptin levels (Kilpeläinen et al., 2016) were mostly population-based or family-based. The three included case-control studies were not specific to leptin levels (NHS: type 2 diabetes and breast cancer; GEMS: dyslipidemia; Health 2000: the condition for cases not mentioned). The EWAS on leptin levels (Yaghootkar et al., 2020) included population-based, family-based and cohort studies. Therefore, it is more reasonable to assume that very low leptin levels (and also very high ones) were not or only partially taken into account in the estimation of genetic associations. In MR, a linear relationship is assumed; non-linear relationships cannot be taken into account with these models. The GWAS on leptin levels and AN, as well as EWAS on leptin levels, do not focus on these phenotypes in childhood. Although some or several of the included studies included children or adolescents, it is only possible to apply our results to children and adolescents if the genetic factors are similar to those in adults. Since most patients with AN are female, GWAS on AN in males are lacking. It is possible that the effects of leptin levels on the risk of AN differ between the sexes.

Two-sample MR makes the assumption of independent samples. Violation of this assumption leads to inflated type 1 error and biased effect estimates (Burgess et al., 2016). In a simulation analysis for binary outcomes with sample overlap in the controls - which would be theoretically possible for our study - there were no detectable biases in the IV estimates, not even with extremely weak instruments, and no inflation of the type 1 error (Burgess et al., 2016).

## Conclusion

Our MR analysis provides support for a causal effect of lower leptin levels on a higher risk of AN. This holds up upon use of data based on leptin levels in females only. This conclusion is based on the analyses of GWAS data, the analyses with EWAS data did not implicate a causal effect. AN itself has no causal effect on leptin levels, although starvation induced hypoleptinemia characterizes patients with acute AN. Apart from a genetic predisposition to lower leptin levels representing a risk factor for the development of AN, the same predisposition may also contribute to the slow recovery and the high risk of relapse.

## Websites

https://ldlink.nci.nih.gov/?tab=home

https://www.ebi.ac.uk/gwas/downloads/summary-statistics [summary statistics (Yaghootkar et al., 2020)]

http://kp4cd.org/node/184 [summary statistics (Kilpeläinen et al., 2016)]

https://www.med.unc.edu/pgc/download-results [summary statistics (Watson et al., 2019)]

http://www.snipa.org

http://genome.ucsc.edu/index.html

https://www.snpedia.com/

https://www.genecards.org/

https://github.com/bulik/ldsc.

## Data Availability

Publicly available datasets were analyzed in this study. This data can be found here: https://www.ebi.ac.uk/gwas/downloads/summary-statistics; http://kp4cd.org/node/184; https://www.med.unc.edu/pgc/download-results.

## References

[B1] American Psychiatric Association (2013). Diagnostic and Statistical Manual of Mental Disorders (DSM-5®). Washington, DC: American Psychiatric Pub.

[B2] Antel J. Tan S. Grabler M. Ludwig C. Lohkemper D. Brandenburg T. (2021). Rapid Amelioration of Anorexia Nervosa in a Male Adolescent During Metreleptin Treatment Including Recovery from Hypogonadotropic Hypogonadism. Eur. Child Adolesc. Psychiatry, 1–7. 10.1007/s00787-021-01778-7 33966118 PMC8106547

[B3] Arnold M. Raffler J. Pfeufer A. Suhre K. Kastenmuller G. (2015). SNiPA: an Interactive, Genetic Variant-Centered Annotation Browser. Bioinformatics 31 (8), 1334–1336. 10.1093/bioinformatics/btu779 25431330 PMC4393511

[B4] Balligand J. L. Brichard S. M. Brichard V. Desager J. P. Lambert M. (1998). Balligand 1998 Hypoleptinemia in Patients with Anorexia Nervosa Loss of Circadian Rhythm and Unresponsiveness to Short-Term refeeding.Pdf. Eur. J. Endocrinol. 138, 415–420. 9578509 10.1530/eje.0.1380415

[B5] Benbaibeche H. Bounihi A. Koceir E. A. (2021). Leptin Level as a Biomarker of Uncontrolled Eating in Obesity and Overweight. Ir J. Med. Sci. 190 (1), 155–161. 10.1007/s11845-020-02316-1 32681271

[B6] Berthoud H. R. (2011). Metabolic and Hedonic Drives in the Neural Control of Appetite: Who Is the Boss?. Curr. Opin. Neurobiol. 21 (6), 888–896. 10.1016/j.conb.2011.09.004 21981809 PMC3254791

[B7] Bowden J. Davey Smith G. Burgess S. (2015). Mendelian Randomization with Invalid Instruments: Effect Estimation and Bias Detection through Egger Regression. Int. J. Epidemiol. 44 (2), 512–525. 10.1093/ije/dyv080 26050253 PMC4469799

[B8] Bowden J. Davey Smith G. Haycock P. C. Burgess S. (2016). Consistent Estimation in Mendelian Randomization with Some Invalid Instruments Using a Weighted Median Estimator. Genet. Epidemiol. 40 (4), 304–314. 10.1002/gepi.21965 27061298 PMC4849733

[B9] Brion M. J. Shakhbazov K. Visscher P. M. (2013). Calculating Statistical Power in Mendelian Randomization Studies. Int. J. Epidemiol. 42 (5), 1497–1501. 10.1093/ije/dyt179 24159078 PMC3807619

[B10] Bulik-Sullivan B. K. Loh P. R. Finucane H. K. Ripke S. Yang J. Daly M. J. (2015). LD Score Regression Distinguishes Confounding from Polygenicity in Genome-wide Association Studies. Nat. Genet. 47 (3), 291–295. 10.1038/ng.3211 25642630 PMC4495769

[B11] Burgess S. Bowden J. Fall T. Ingelsson E. Thompson S. G. (2017a). Sensitivity Analyses for Robust Causal Inference from Mendelian Randomization Analyses with Multiple Genetic Variants. Epidemiology. 28 (1), 30–42. 10.1097/EDE.0000000000000559 27749700 PMC5133381

[B12] Burgess S. Davey Smith G. Davies N. M. Dudbridge F. Gill D. Glymour M. M. (2019). Guidelines for Performing Mendelian Randomization Investigations. Wellcome Open Res. 4, 186. 10.12688/wellcomeopenres.15555.2 32760811 PMC7384151

[B13] Burgess S. Davies N. M. Thompson S. G. (2016). Bias Due to Participant Overlap in Two-Sample Mendelian Randomization. Genet. Epidemiol. 40 (7), 597–608. 10.1002/gepi.21998 27625185 PMC5082560

[B14] Burgess S. Small D. S. Thompson S. G. (2017b). A Review of Instrumental Variable Estimators for Mendelian Randomization. Stat. Methods Med. Res. 26 (5), 2333–2355. 10.1177/0962280215597579 26282889 PMC5642006

[B15] Burgess S. Thompson S. G. (2017). Interpreting Findings from Mendelian Randomization Using the MR-Egger Method. Eur. J. Epidemiol. 32 (5), 377–389. 10.1007/s10654-017-0255-x 28527048 PMC5506233

[B16] Burgess S. Timpson N. J. Ebrahim S. Davey Smith G. (2015). Mendelian Randomization: where Are We Now and where Are We Going? Int. J. Epidemiol. 44 (2), 379–388. 10.1093/ije/dyv108 26085674

[B17] Dallner O. S. Marinis J. M. Lu Y. H. Birsoy K. Werner E. Fayzikhodjaeva G. (2019). Dysregulation of a Long Noncoding RNA Reduces Leptin Leading to a Leptin-Responsive Form of Obesity. Nat. Med. 25 (3), 507–516. 10.1038/s41591-019-0370-1 30842678

[B18] Davey Smith G. Davies N. M. Dimou N. Egger M. Gallo V. Golub R. (2019). STROBE-MR: Guidelines for Strengthening the Reporting of Mendelian Randomization Studies. PeerJ Preprints 7, e27857v1. 10.7287/peerj.preprints.27857v1

[B19] Davey Smith G. Ebrahim S. (2003). Mendelian Randomization: Can Genetic Epidemiology Contribute to Understanding Environmental Determinants of Disease?. Int. J. Epidemiol. 32, 1–22. 10.1093/ije/dyg070 12689998

[B20] Davey Smith G. Ebrahim S. (2008). “Mendelian Randomization: Genetic Variants as Instruments for Strengthening Causal Inference in Observational Studies,” in National Research Council (US) Committee on Advances in Collecting and Utilizing Biological Indicators and Genetic Information in Social Science Surveys. Biosocial Surveys. (Washington (DC): National Academies Press (US)).

[B21] Davey Smith G. Ebrahim S. (2005). What Can Mendelian Randomisation Tell Us about Modifiable Behavioural and Environmental Exposures. BMJ 330, 1076–1079. 10.1136/bmj.330.7499.1076 15879400 PMC557238

[B22] Davis C. Kennedy S. H. Ravelski E. Dionne M. (1994). The Role of Physical Activity in the Development and Maintenance of Eating Disorders. Psychol. Med. 24 (4), 957–967. 10.1017/s0033291700029044 7892363

[B23] de la Brousse F. C. Shan B. Chen J. L. (1996). Identification of the Promoter of the Mouse Obese Gene. Proc. Natl. Acad. Sci. 93 (9), 4096–4101. 10.1073/pnas.93.9.4096 8633022 PMC39493

[B25] Ebrahim S. Davey Smith G. (2008). Mendelian Randomization: Can Genetic Epidemiology Help Redress the Failures of Observational Epidemiology? Hum. Genet. 123 (1), 15–33. 10.1007/s00439-007-0448-6 18038153

[B26] Egecioglu E. Skibicka K. P. Hansson C. Alvarez-Crespo M. Friberg P. A. Jerlhag E. (2011). Hedonic and Incentive Signals for Body Weight Control. Rev. Endocr. Metab. Disord. 12 (3), 141–151. 10.1007/s11154-011-9166-4 21340584 PMC3145094

[B27] Farooqi I. S. Bullmore E. Keogh J. Gillard J. O'Rahilly S. Fletcher P. C. (2007). Leptin Regulates Striatal Regions and Human Eating Behavior. Science 317 (5843), 1355. 10.1126/science.1144599 17690262 PMC3838941

[B28] Fernandes M. F. Matthys D. Hryhorczuk C. Sharma S. Mogra S. Alquier T. (2015). Leptin Suppresses the Rewarding Effects of Running *via* STAT3 Signaling in Dopamine Neurons. Cell Metab. 22 (4), 741–749. 10.1016/j.cmet.2015.08.003 26341832

[B29] Grinspoon S. Gulick T. Askari H. Landt M. Lee K. Anderson E. (1996). Serum Leptin Levels in Women with Anorexia Nervosa. J. Clin. Endocrinol. Metab. 81 (11), 3861–3863. 10.1210/jcem.81.11.8923829 8923829

[B30] Hara K. Fujita H. Johnson T. A. Yamauchi T. Yasuda K. Horikoshi M. (2014). Genome-wide Association Study Identifies Three Novel Loci for Type 2 Diabetes. Hum. Mol. Genet. 23 (1), 239–246. 10.1093/hmg/ddt399 23945395

[B31] Hartwig F. P. Davey Smith G. Bowden J. (2017). Robust Inference in Summary Data Mendelian Randomization *via* the Zero Modal Pleiotropy assumption. Int. J. Epidemiol. 46 (6), 1985–1998. 10.1093/ije/dyx102 29040600 PMC5837715

[B32] Hartwig F. P. Davies N. M. Hemani G. Davey Smith G. (2016). Two-sample Mendelian Randomization: Avoiding the Downsides of a Powerful, Widely Applicable but Potentially Fallible Technique. Int. J. Epidemiol. 45 (6), 1717–1726. 10.1093/ije/dyx028 28338968 PMC5722032

[B33] Haycock P. C. Burgess S. Wade K. H. Bowden J. Relton C. Davey Smith G. (2016). Best (But Oft-Forgotten) Practices: the Design, Analysis, and Interpretation of Mendelian Randomization Studies. Am. J. Clin. Nutr. 103 (4), 965–978. 10.3945/ajcn.115.118216 26961927 PMC4807699

[B34] Hebebrand J. Albayrak O. Adan R. Antel J. Dieguez C. de Jong J. (2014). Eating Addiction", rather Than "food Addiction", Better Captures Addictive-like Eating Behavior. Neurosci. Biobehav Rev. 47, 295–306. 10.1016/j.neubiorev.2014.08.016 25205078

[B35] Hebebrand J. Blum W. F. Barth N. Coners H. Englaro P. Juul A. (1997). Leptin Levels in Patients with Anorexia Nervosa Are Reduced in the Acute Stage and Elevated upon Short-Term Weight Restoration. Mol. Psychiatry 2, 330–334. 9246674 10.1038/sj.mp.4000282

[B36] Hebebrand J. Bulik C. M. (2011). Critical Appraisal of the Provisional DSM-5 Criteria for Anorexia Nervosa and an Alternative Proposal. Int. J. Eat. Disord. 44 (8), 665–678. 10.1002/eat.20875 22072403

[B37] Hebebrand J. Milos G. Wabitsch M. Teufel M. Fuhrer D. Buhlmeier J. (2019). Clinical Trials Required to Assess Potential Benefits and Side Effects of Treatment of Patients with Anorexia Nervosa with Recombinant Human Leptin. Front. Psychol. 10, 769. 10.3389/fpsyg.2019.00769 31156489 PMC6533856

[B38] Hebebrand J. van der Heyden J. Devos R. Köpp W. Herpertz S. Remschmidt H. (1995). Plasma concentrations of obese protein in anorexia nervosa *Lancet* 346 (8990), 1624–1625. 10.1016/s0140-6736(95)91955-4 7500762

[B39] Hellström L. Wahrenberg H. Hruska K. Reynisdottir S. Arner P. (2000). Mechanisms behind Gender Differences in Circulating Leptin Levels. J. Intern. Med. 247, 457–462. 10.1046/j.1365-2796.2000.00678.x 10792559

[B40] Hemani G. Zheng J. Elsworth B. Wade K. H. Haberland V. Baird D. (2018). The MR-Base Platform Supports Systematic Causal Inference across the Human Phenome. Elife 7. 10.7554/eLife.34408 PMC5976434 29846171

[B41] Higgs S. Spetter M. S. Thomas J. M. Rotshtein P. Lee M. Hallschmid M. (2017). Interactions between Metabolic, Reward and Cognitive Processes in Appetite Control: Implications for Novel Weight Management Therapies. J. Psychopharmacol. 31 (11), 1460–1474. 10.1177/0269881117736917 29072515 PMC5700796

[B42] Hinney A. Volckmar A. L. Antel J. (2014). Genes and the Hypothalamic Control of Metabolism in Humans. Best Pract. Res. Clin. Endocrinol. Metab. 28 (5), 635–647. 10.1016/j.beem.2014.04.007 25256760

[B43] Hübel C. Gaspar H. A. Coleman J. R. I. Hanscombe K. B. Purves K. Prokopenko I. (2019). Genetic Correlations of Psychiatric Traits with Body Composition and Glycemic Traits Are Sex- and Age-dependent. Nat. Commun. 10 (1), 5765. 10.1038/s41467-019-13544-0 31852892 PMC6920448

[B44] Katikireddi S. V. Green M. J. Taylor A. E. Davey Smith G. Munafo M. R. (2018). Assessing Causal Relationships Using Genetic Proxies for Exposures: an Introduction to Mendelian Randomization. Addiction 113 (4), 764–774. 10.1111/add.14038 28921935 PMC5873430

[B45] Kilpeläinen T. O. Carli J. F. Skowronski A. A. Sun Q. Kriebel J. Feitosa M. F. (2016). Genome-wide Meta-Analysis Uncovers Novel Loci Influencing Circulating Leptin Levels. Nat. Commun. 7, 10494. 10.1038/ncomms10494 26833098 PMC4740377

[B46] Kim J. Y. Tillison K. Smas C. M. (2004). Cloning, Expression, and Differentiation-dependent Regulation of SMAF1 in Adipogenesis. Biochem. Biophysical Res. Commun. 326 (1), 36–44. 10.1016/j.bbrc.2004.10.200 15567149

[B47] König I. R. Greco F. M. D. (2018). Mendelian Randomization: Progressing towards Understanding Causality. Ann. Neurol. 84 (2), 176–177. 10.1002/ana.25293 30014502 PMC6221001

[B48] Lavender J. M. Wonderlich S. A. Engel S. G. Gordon K. H. Kaye W. H. Mitchell J. E. (2015). Dimensions of Emotion Dysregulation in Anorexia Nervosa and Bulimia Nervosa: A Conceptual Review of the Empirical Literature. Clin. Psychol. Rev. 40, 111–122. 10.1016/j.cpr.2015.05.010 26112760 PMC4537813

[B49] Lawlor D. A. Harbord R. M. Sterne J. A. Timpson N. Davey Smith G. (2008). Mendelian Randomization: Using Genes as Instruments for Making Causal Inferences in Epidemiology. Stat. Med. 27 (8), 1133–1163. 10.1002/sim.3034 17886233

[B50] Lee J. J. Wedow R. Okbay A. Kong E. Maghzian O. Zacher M. (2018). Gene Discovery and Polygenic Prediction from a Genome-wide Association Study of Educational Attainment in 1.1 Million Individuals. Nat. Genet. 50 (8), 1112–1121. 10.1038/s41588-018-0147-3 30038396 PMC6393768

[B51] Li D. Chang X. Connolly J. J. Tian L. Liu Y. Bhoj E. J. (2017). A Genome-wide Association Study of Anorexia Nervosa Suggests a Risk Locus Implicated in Dysregulated Leptin Signaling. Sci. Rep. 7 (1), 3847. 10.1038/s41598-017-01674-8 28630421 PMC5476671

[B52] Lichtenstein M. B. Andries A. Hansen S. Frystyk J. Støving R. K. (2015). Exercise Addiction in Men Is Associated with Lower Fat-Adjusted Leptin Levels. Clin. J. Sport Med. 25 (2), 138–143. 10.1097/jsm.0000000000000110 24926913

[B53] Lu X. Y. (2007). The Leptin Hypothesis of Depression: a Potential Link between Mood Disorders and Obesity?. Curr. Opin. Pharmacol. 7 (6), 648–652. 10.1016/j.coph.2007.10.010 18032111 PMC2677994

[B54] Mantzoros C. S. Magkos F. Brinkoetter M. Sienkiewicz E. Dardeno T. A. Kim S. Y. (2011). Leptin in Human Physiology and Pathophysiology. Am. J. Physiol. Endocrinol. Metab. 301 (4), E567–E584. 10.1152/ajpendo.00315.2011 21791620 PMC3191548

[B55] Margetic S. Gazzola C. Pegg G. G. Hill R. A. (2002). Leptin: a Review of its Peripheral Actions and Interactions. Int. J. Obes. 26 (11), 1407–1433. 10.1038/sj.ijo.0802142 12439643

[B56] Mason M. M. He Y. Chen H. Quon M. J. Reitman M. (1998). Regulation of Leptin Promoter Function by Sp1. C/EBP, a novel Factor *Endocrinol.* 139 (3), 1013–1022. 10.1210/endo.139.3.5792 9492033

[B57] Miller K. K. (2011). Endocrine Dysregulation in Anorexia Nervosa Update. J. Clin. Endocrinol. Metab. 96 (10), 2939–2949. 10.1210/jc.2011-1222 21976742 PMC3200238

[B58] Miller R. Tanofsky-Kraff M. Shomaker L. B. Field S. E. Hannallah L. Reina S. A. (2014). Serum Leptin and Loss of Control Eating in Children and Adolescents. Int. J. Obes. (Lond) 38 (3), 397–403. 10.1038/ijo.2013.126 23835660 PMC3900593

[B59] Milos G. Antel J. Kaufmann L. K. Barth N. Koller A. Tan S. (2020). Short-term Metreleptin Treatment of Patients with Anorexia Nervosa: Rapid On-Set of Beneficial Cognitive, Emotional, and Behavioral Effects. Transl Psychiatry 10 (1), 303. 10.1038/s41398-020-00977-1 32855384 PMC7453199

[B60] Misra M. Klibanski A. (2014). Endocrine Consequences of Anorexia Nervosa. Lancet Diabetes Endocrinol. 2 (7), 581–592. 10.1016/S2213-8587(13)70180-3 24731664 PMC4133106

[B61] Morrison C. D. (2009). Leptin Signaling in Brain: A Link between Nutrition and Cognition?. Biochim. Biophys. Acta 1792 (5), 401–408. 10.1016/j.bbadis.2008.12.004 19130879 PMC2670357

[B62] Munzberg H. Heymsfield S. B. (2019). New Insights into the Regulation of Leptin Gene Expression. Cel Metab 29 (5), 1013–1014. 10.1016/j.cmet.2019.04.005 PMC7346278 31067443

[B63] Nicholls D. E. Lynn R. Viner R. M. (2011). Childhood Eating Disorders: British National Surveillance Study. Br. J. Psychiatry 198 (4), 295–301. 10.1192/bjp.bp.110.081356 21972279

[B64] Peelman F. Zabeau L. Moharana K. Savvides S. N. Tavernier J. (2014). 20 Years of Leptin: Insights into Signaling Assemblies of the Leptin Receptor. J. Endocrinol. 223 (1), T9–T23. 10.1530/JOE-14-0264 25063754

[B65] Pierce B. L. Burgess S. (2013). Efficient Design for Mendelian Randomization Studies: Subsample and 2-sample Instrumental Variable Estimators. Am. J. Epidemiol. 178 (7), 1177–1184. 10.1093/aje/kwt084 23863760 PMC3783091

[B66] Raitoharju E. Seppälä I. Oksala N. Lyytikäinen L.-P. Raitakari O. Viikari J. (2014). Blood microRNA Profile Associates with the Levels of Serum Lipids and Metabolites Associated with Glucose Metabolism and Insulin Resistance and Pinpoints Pathways Underlying Metabolic Syndrome: The Cardiovascular Risk in Young Finns Study. Mol. Cell Endocrinol. 391 (1), 41–49. 10.1016/j.mce.2014.04.013 24784704

[B67] Rees J. M. B. Wood A. M. Dudbridge F. Burgess S. (2019). Robust Methods in Mendelian Randomization *via* Penalization of Heterogeneous Causal Estimates. PLoS One 14 (9), e0222362. 10.1371/journal.pone.0222362 31545794 PMC6756542

[B68] Steinhausen H. C. Jensen C. M. (2015). Time Trends in Lifetime Incidence Rates of First-Time Diagnosed Anorexia Nervosa and Bulimia Nervosa across 16 Years in a Danish Nationwide Psychiatric Registry Study. Int. J. Eat. Disord. 48 (7), 845–850. 10.1002/eat.22402 25809026

[B69] Swerdlow D. I. Kuchenbaecker K. B. Shah S. Sofat R. Holmes M. V. White J. (2016). Selecting Instruments for Mendelian Randomization in the Wake of Genome-wide Association Studies. Int. J. Epidemiol. 45 (5), 1600–1616. 10.1093/ije/dyw088 27342221 PMC5100611

[B70] Thompson-Brenner H. Eddy K. T. Satir D. A. Boisseau C. L. Westen D. (2008). Personality Subtypes in Adolescents with Eating Disorders: Validation of a Classification Approach. J. Child. Psychol. Psychiatry 49 (2), 170–180. 10.1111/j.1469-7610.2007.01825.x 18093115

[B71] Vayalapalli A. Deshpande R. Vayalapalli A. (2018). The Relationship between Exercise and Athletes to the Pathogenesis and Recovery from Anorexia Nervosa. Int. J. Neurosci. Behav. Sci. 6 (2), 17–21. 10.13189/ijnbs.2018.060201

[B72] Verbanck M. Chen C. Y. Neale B. Do R. (2018). Detection of Widespread Horizontal Pleiotropy in Causal Relationships Inferred from Mendelian Randomization between Complex Traits and Diseases. Nat. Genet. 50 (5), 693–698. 10.1038/s41588-018-0099-7 29686387 PMC6083837

[B73] Volkow N. D. Wang G. J. Baler R. D. (2011). Reward, Dopamine and the Control of Food Intake: Implications for Obesity. Trends Cogn. Sci. 15 (1), 37–46. 10.1016/j.tics.2010.11.001 21109477 PMC3124340

[B74] Watson H. J. Yilmaz Z. Thornton L. M. Hubel C. Coleman J. R. I. Gaspar H. A. (2019). Genome-wide Association Study Identifies Eight Risk Loci and Implicates Metabo-Psychiatric Origins for Anorexia Nervosa. Nat. Genet. 51 (8), 1207–1214. 10.1038/s41588-019-0439-2 31308545 PMC6779477

[B75] Wonderlich S. A. Lilenfeld L. R. Riso L. P. Engel S. Mitchell J. E. (2005). Personality and Anorexia Nervosa. Int. J. Eat. Disord. 37 (Suppl. l), S68–S71. discussion S87-69. 10.1002/eat.20120 15852324

[B76] Yaghootkar H. Zhang Y. Spracklen C. N. Kilpeläinen T. O. Huang L. O. Bradfield J. (2020). Genetic Studies of Leptin Concentrations Implicate Leptin in the Regulation of Early Adiposity. Diabetes 69 (12), 2806–2818. 10.2337/db20-0070 32917775 PMC7679778

[B77] Zhao J.-J. Yang J. Lin J. Yao N. Zhu Y. Zheng J. (2009). Identification of miRNAs Associated with Tumorigenesis of Retinoblastoma by miRNA Microarray Analysis. Child's Nervous Syst. 25 (1), 13–20. 10.1007/s00381-008-0701-x 18818933

[B78] Zhao Q. Wang J. Hemani G. Bowden J. Small D. S. (2020). Statistical Inference in Two-Sample Summary-Data Mendelian Randomization Using Robust Adjusted Profile Score. Ann. Stat. 48 (3). 10.1214/19-aos1866

[B79] Zhao S. S. Mackie S. L. Zheng J. (2021). Why Clinicians Should Know about Mendelian Randomization. Rheumatology 60, 1577–1579. 10.1093/rheumatology/keab007 33493347

[B80] Zou X. Zhong L. Zhu C. Zhao H. Zhao F. Cui R. (2019). Role of Leptin in Mood Disorder and Neurodegenerative Disease. Front. Neurosci. 13, 378. 10.3389/fnins.2019.00378 31130833 PMC6510114

