# Supplementary material for: Suggestive Evidence for Causal Effect of Leptin Levels on Risk for Anorexia Nervosa: Results of a Mendelian Randomization Study
Source: Front Genet. 2021 Sep 14;12:733606. doi: 10.3389/fgene.2021.733606 (PMC8476861; doi:10.3389/fgene.2021.733606)
Supplement: Supplementary file 1 [file DataSheet1.PDF]

Supplementary Figures and Tables

Suggestive Evidence for Causal Effect of Leptin Levels on Risk for Anorexia Nervosa: Results of a Mendelian Randomization Study

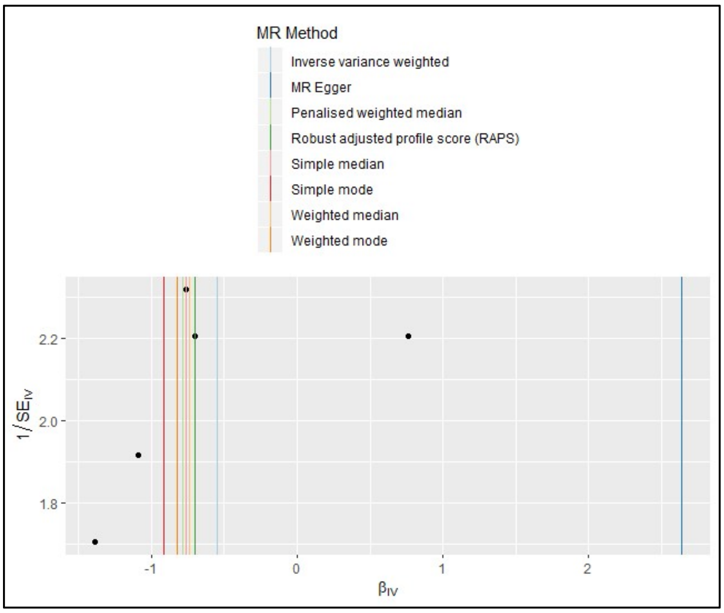

Figure S1. Funnel plot: Mendelian randomization (MR) analyses with leptin levels (Kilpeläinen et al., 2016) as exposure and risk for anorexia nervosa (AN) (Watson et al., 2019) as outcome

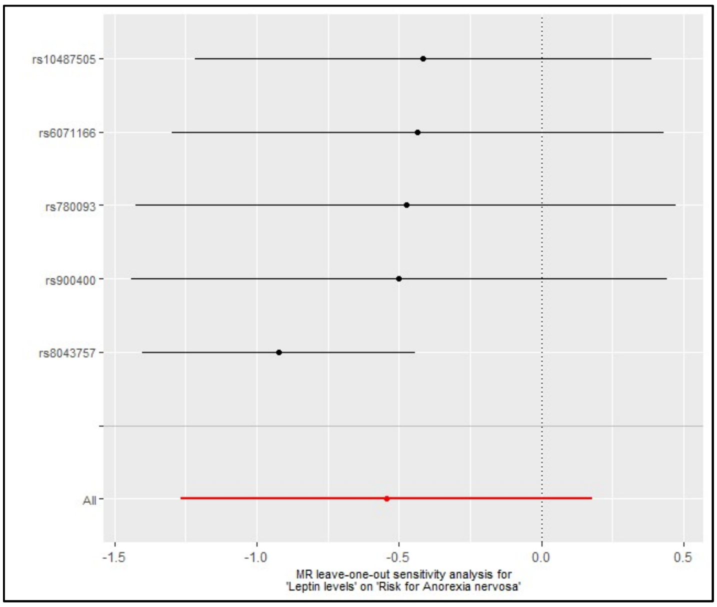

Figure S2. Leave out analyses using IVW method: Mendelian randomization (MR) analyses with leptin levels (Kilpeläinen et al., 2016) as exposure and risk for anorexia nervosa (Watson et al., 2019) as outcome

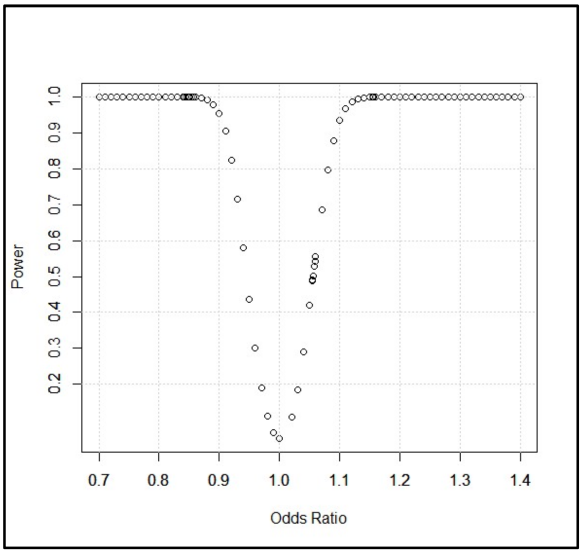

Figure S3. Calculated power to detect a true causal effect: power of 0.80 for OR=0.92/1.08; the following data were used for the calculation: K=0.234 (AN), N=72517 (AN), R^2_{xy}=0.097 (GWAS on leptin levels (Kilpeläinen et al. (2016), calculated with LDCS tool (Bulik-Sullivan et al., 2015))

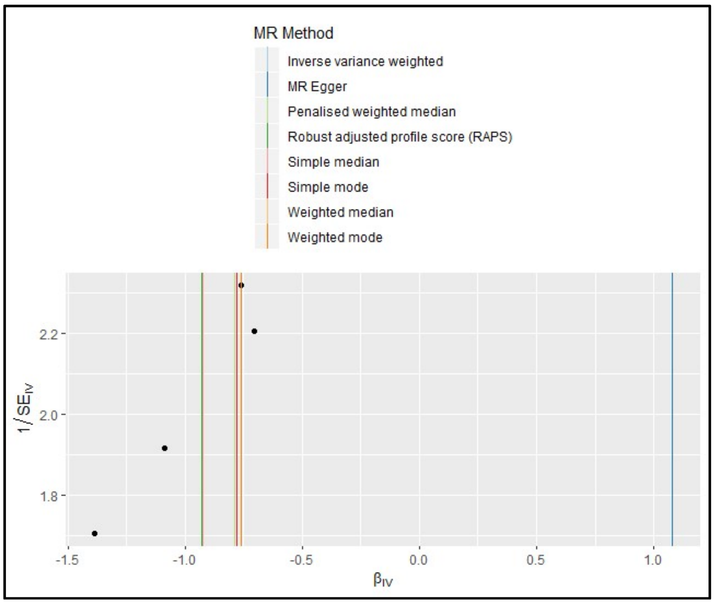

Figure S4. Funnel plot: Mendelian randomization (MR) analyses with leptin levels (Kilpeläinen et al., 2016) as exposure and risk for anorexia nervosa (Watson et al., 2019) as outcome. The BMI-associated SNP rs8043757 was excluded

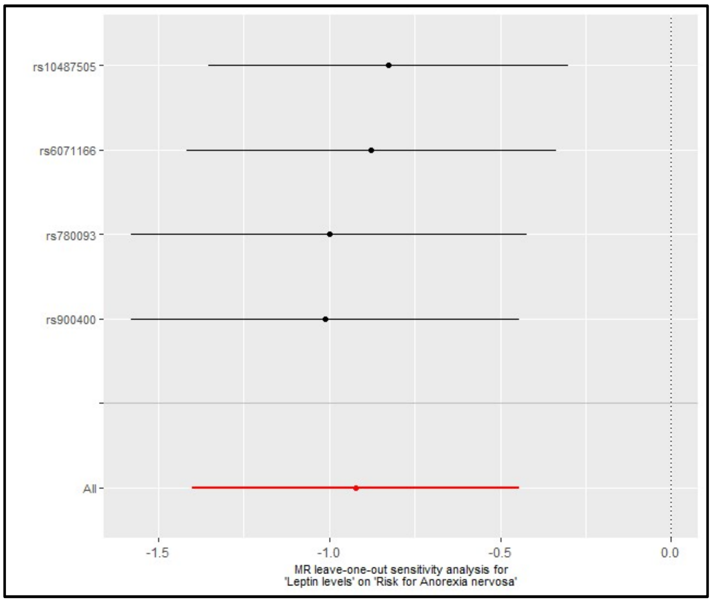

Figure S5. Leave out analyses using IVW method: Mendelian randomization (MR) analyses with leptin levels (Kilpeläinen et al., 2016) as exposure and risk for anorexia nervosa (Watson et al., 2019) as outcome. The BMI-associated SNP rs8043757 was excluded

Table S1. Single nucleotide polymorphisms (SNPs) associated with leptin levels (unadjusted and adjusted for BMI, (µg/ml logarithmically transformed)) in females (Kilpeläinen et al., 2016) and their association with the risk for anorexia nervosa (AN) (OR is transformed to beta) (Watson et al., 2019)

SNP	Nearest Gene	Chr	EA	OA	Leptin levels unadjusted for BMI*					Leptin levels adjusted for BMI*			Anorexia nervosa		
					EAF	b	se	p	F	b	se	p	b	se	p
rs10487505	*LEP*	7	G	C	0.50	0.033	0.0071	4.1E-06	21.60	*0.033*	*0.0054*	*1.2E-09*	-0.032	0.0135	0.0184
rs6071166	*SLC32A1*	20	C	A	0.37	0.031	0.0074	3.7E-05	17.55	*0.025*	*0.0057*	*7.5E-06*	-0.029	0.0141	0.0378
rs780093	*GCKR*	2	C	T	0.61	0.033	0.0067	7.9E-07	24.26	*0.025*	*0.0050*	*1.0E-06*	-0.024	0.0138	0.0778
rs8043757	*FTO*	16	T	A	0.40	0.023	0.0066	4.5E-04	12.14	*-0.008*	*0.0050*	*1.3E-01*	0.023	0.0136	0.0925
rs900400	*CCNL1*	3	T	C	0.60	0.032	0.0068	3.3E-06	22.15	*0.023*	*0.0051*	*9.4E-06*	-0.021	0.0136	0.1204

* Effect sizes have been calculated for subjects with European ancestry
Font in italic: these effect sizes were used to assess the effect of BMI on leptin levels
Chr: chromosome, EA: effect allele; OA: other allele; EAF: effect allele frequency; b: effect size of EA; se: standard error; p: p-value; F: F-test

Table S2. Results of single SNP Mendelian randomization (MR) analyses and the overall causal effect of unadjusted leptin levels in females (Kilpeläinen et al., 2016) on the risk of anorexia nervosa (Watson et al., 2019) calculated using different methods. The beta estimates the change in risk for AN (OR is transformed to beta) per change of 1 unit of leptin concentration (log-transformed µg/ml).

SNP	b	se	p	Lower 95% CI	Upper 95% CI
rs10487505	-0.967	0.409	**0.018**		
rs6071166	-0.948	0.455	**0.037**		
rs780093	-0.736	0.418	0.078		
rs8043757	0.991	0.591	0.094		
rs900400	-0.659	0.425	0.121		
MR Egger	-5.145	1.613	0.050	-8.307	-1.984
Inverse variance weighted	-0.617	0.296	**0.037**	-1.198	-0.037
Simple median	-0.736	0.275	**7.39E-03**	-1.275	-0.198
Weighted median	-0.767	0.279	**6.01E-03**	-1.315	-0.220
Penalised weighted median	-0.814	0.278	**3.37E-03**	-1.359	-0.270
Simple mode	-0.833	0.289	**0.045**	-1.399	-0.267
Weighted mode	-0.814	0.299	0.053	-1.401	-0.228
Robust adjusted profile score (RAPS)	-0.743	0.240	**1.99E-03**	-1.214	-0.272

Horizontal pleiotropy: MR Eggers intercept: 0.140. se=0.049. p=0.066; MR PRESSO no outlier
Heterogeneity test: MR Egger Q(df=3)=0.748. p=0.862; IVW Q(df=4)=8.750. p=0.068
b: effect size; se: standard error; p: p-value

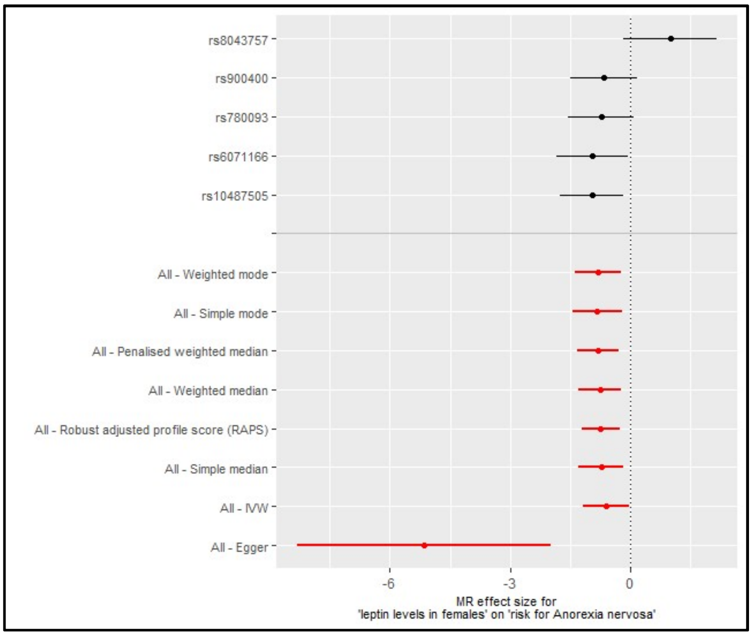

Figure S6. Results of the single and multi-SNP analyses for the SNP effect of leptin levels (Kilpeläinen et al., 2016)on risk for anorexia nervosa (Watson et al., 2019). The black lines visualize the results of single SNP analyses; the red lines visualize the results of the multi SNP analysis

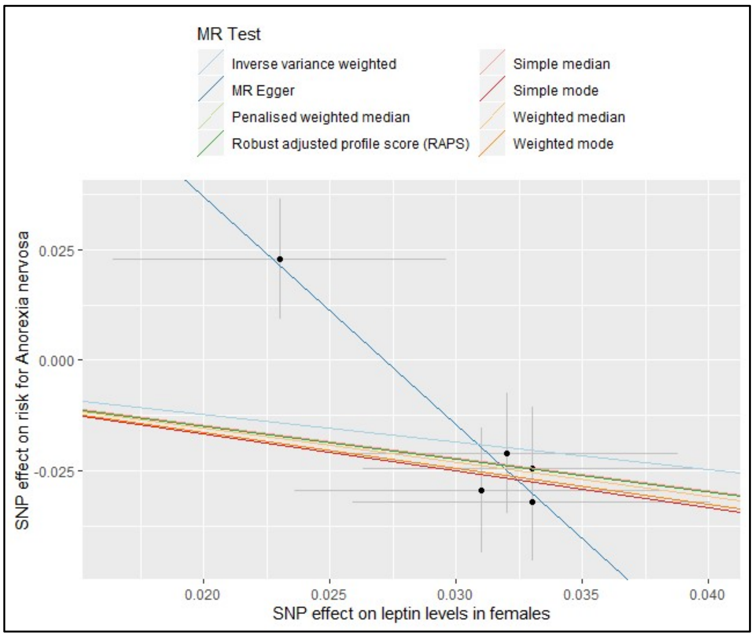

Figure S7. Scatter plots of genetic associations with leptin levels in females (Kilpeläinen et al., 2016) against risk for anorexia nervosa using different Mendelian randomization (MR) methods. The slopes of each line represent the causal association for each method.

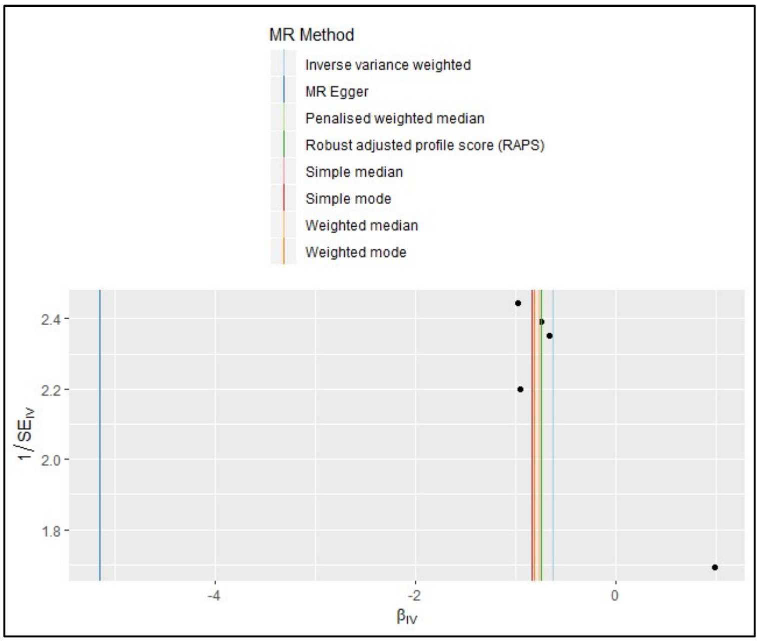

Figure S8. Funnel plot: Mendelian randomization (MR) analyses with leptin levels in female (Kilpeläinen et al. 2016) as exposure and risk for anorexia nervosa (Watson et al., 2019) as outcome

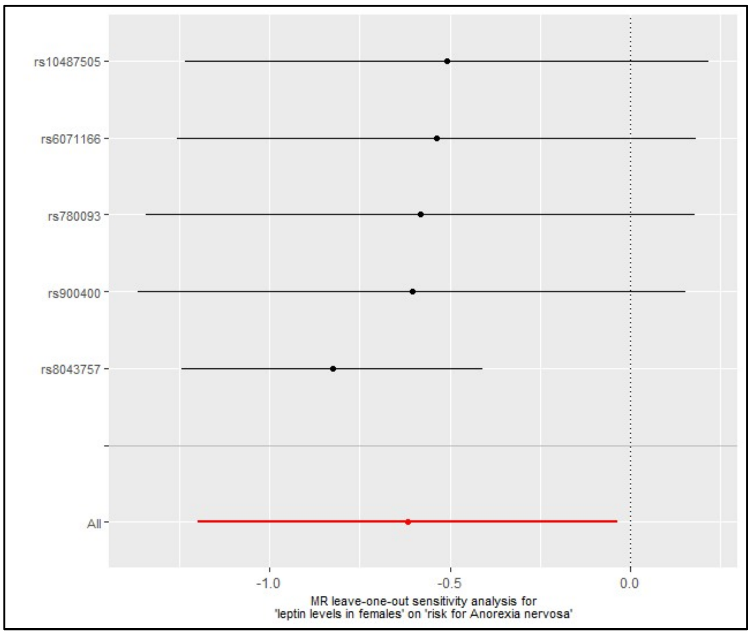

Figure S9. Leave out analyses using IVW method: Mendelian randomization (MR) analyses with leptin levels in females (Kilpeläinen et al., 2016) as exposure and risk for anorexia nervosa (Watson et al., 2019) as outcome.

Table S3. Results of single SNP MR analyses and the overall causal effect of leptin levels in females (Kilpeläinen et al., 2016) on the risk of Anorexia nervosa (Watson et al., 2019) calculated using different methods. The BMI-associated SNP rs8043757 (*FTO*) was excluded. The beta estimates the change in risk for AN (OR is transformed to beta) per change of 1 unit of leptin concentration (log-transformed µg/ml).

SNP	b	se	p	Lower 95% CI	Upper 95% CI
**rs10487505**	-0.967	0.409	**0.018**		
**rs6071166**	-0.948	0.455	**0.037**		
rs780093	-0.736	0.418	0.078		
rs900400	-0.659	0.425	0.121		
MR Egger	-0.182	8.383	0.985	-16.613	16.249
Inverse variance weighted	-0.826	0.213	**1.05E-04**	-1.243	-0.409
Simple median	-0.842	0.277	**2.36E-03**	-1.385	-0.299
Weighted median	-0.822	0.265	**1.88E-03**	-1.341	-0.304
Penalised weighted median	-0.822	0.266	**2.02E-03**	-1.345	-0.300
Simple mode	-0.937	0.329	0.065	-1.582	-0.292
Weighted mode	-0.717	0.353	0.136	-1.410	-0.024
Robust adjusted profile score (RAPS)	-0.828	0.238	**5.15E-04**	-1.295	-0.361

Horizontal pleiotropy: Eggers intercept= -0.021. se=0.271. p=0.946; MR PRESSO no outlier
Heterogeneity test: MR Egger Q(df=2)=0.384. p=0.825; IVW Q(df=3)=0.390. p=0.942
b: effect size; se: standard error; p: p-value

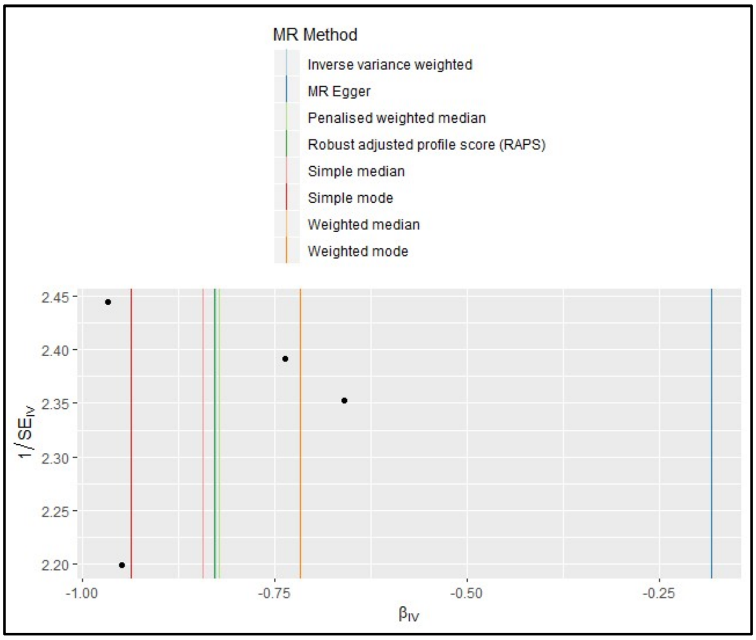

Figure S10. Funnel plot: Mendelian randomization (MR) analyses with leptin levels in female (Kilpeläinen et al., 2016) exposure and risk for anorexia nervosa (Watson et al., 2019) as outcome. The BMI-associated SNP rs8043757 (*FTO*) was excluded.

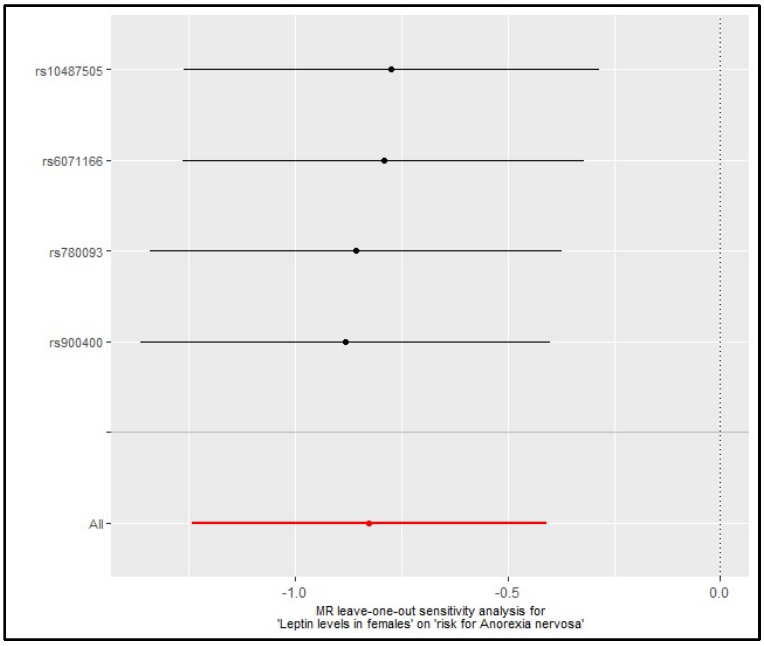

Figure S11. Leave out analyses using IVW method: Mendelian randomization (MR) analyses with leptin levels in females (Kilpeläinen et al., 2016) as exposure and risk for anorexia nervosa (Watson et al., 2019) as outcome. The BMI-associated SNP rs8043757 (*FTO*) was excluded.

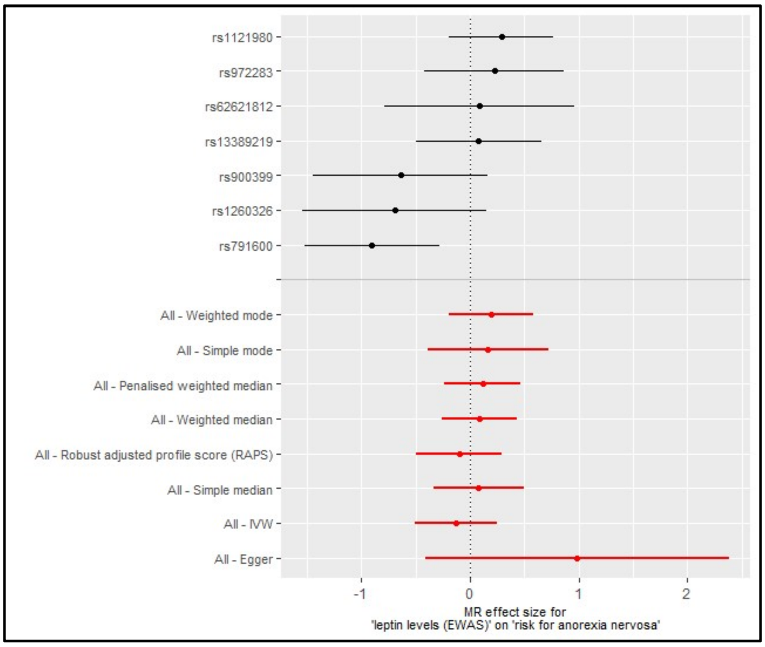

Figure 12. Results of the single and multi-SNP analyses for the SNP effect of leptin levels (Yaghootkar, Zhang, C.N., .., & Kilpeläinen, 2020) on risk for anorexia nervosa (Watson et al., 2019). The black lines visualize the results of single SNP analyses; the red lines visualize the results of the multi SNP analysis

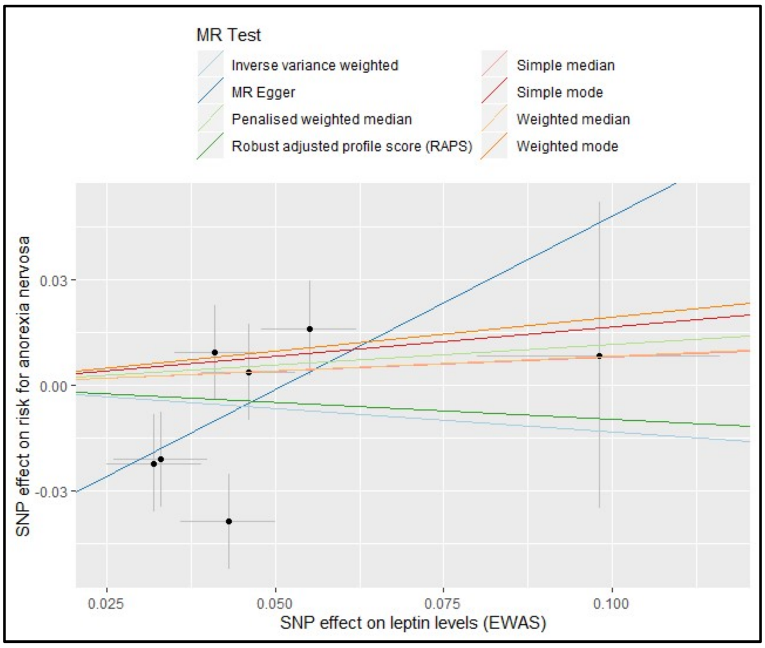

Figure S13. Scatter plots of genetic associations with leptin levels (Yaghootkar et al., 2020) against risk for anorexia nervosa (Watson et al., 2019) using different Mendelian randomization (MR) methods. The slopes of each line represent the causal association for each method.

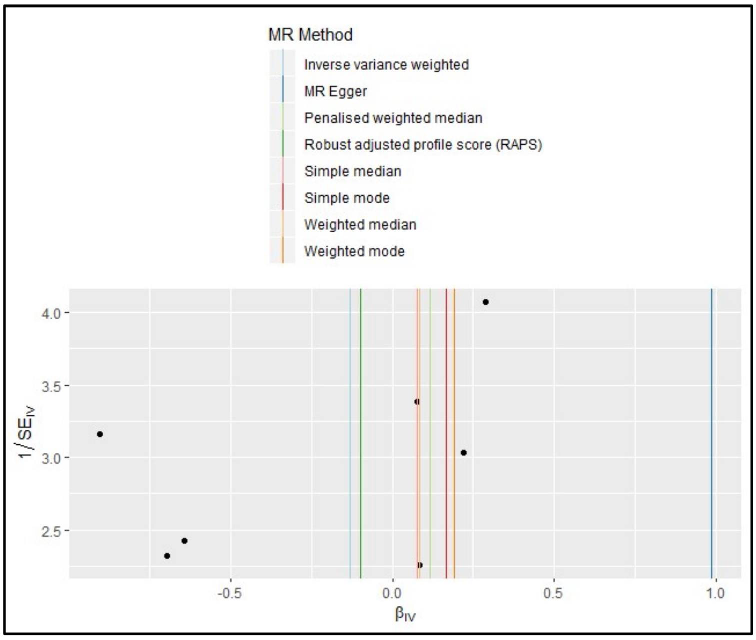

Figure S14. Funnel plot: Mendelian randomization (MR) analyses with leptin levels (European ancestry, additive model) (Yaghootkar et al., 2020) as exposure and anorexia nervosa (Watson et al., 2019) as outcome

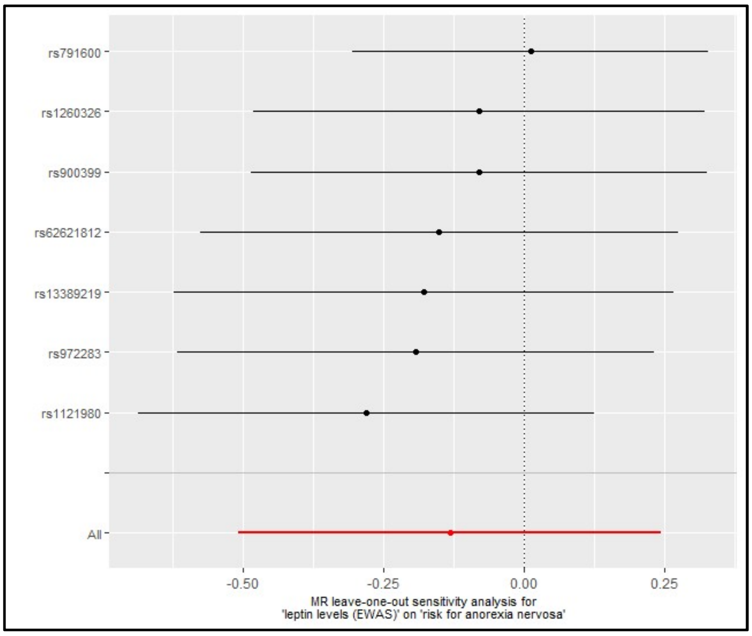

Figure S15. Leave out analyses using IVW method: Mendelian randomization (MR) analyses with leptin levels (Yaghootkar et al., 2020) as exposure and risk for anorexia nervosa (Watson et al., 2019) as outcome

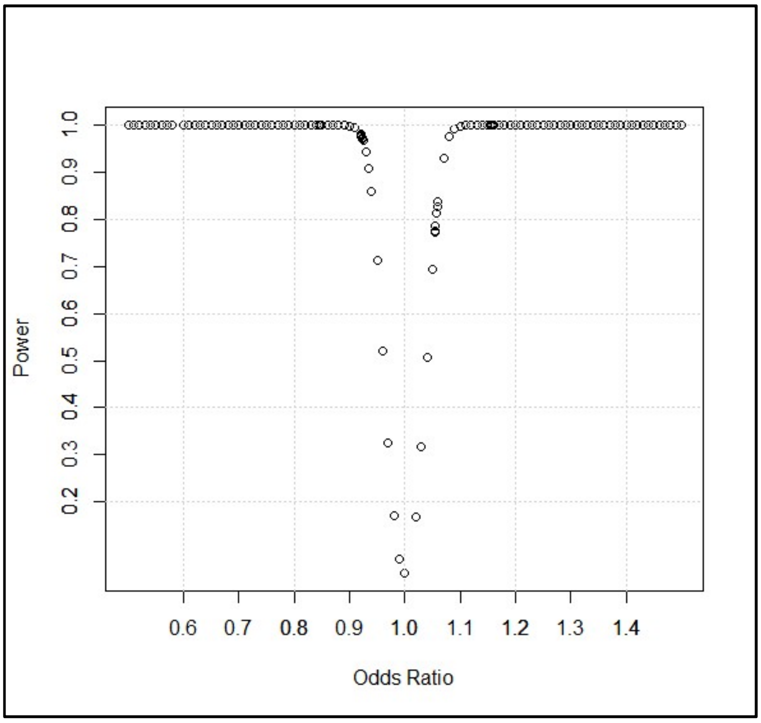

Figure 16. Calculated power to detect a true causal effect: power of 0.80 for OR=0.944/1.057; the following data were used for the calculation: K=0.234 (AN), N=72517 (AN), R^2_{xy}=0.1913 (EWAS on leptin levels (Yaghootkar et al., 2020), calculated with LDCS tool (Bulik-Sullivan et al., 2015))

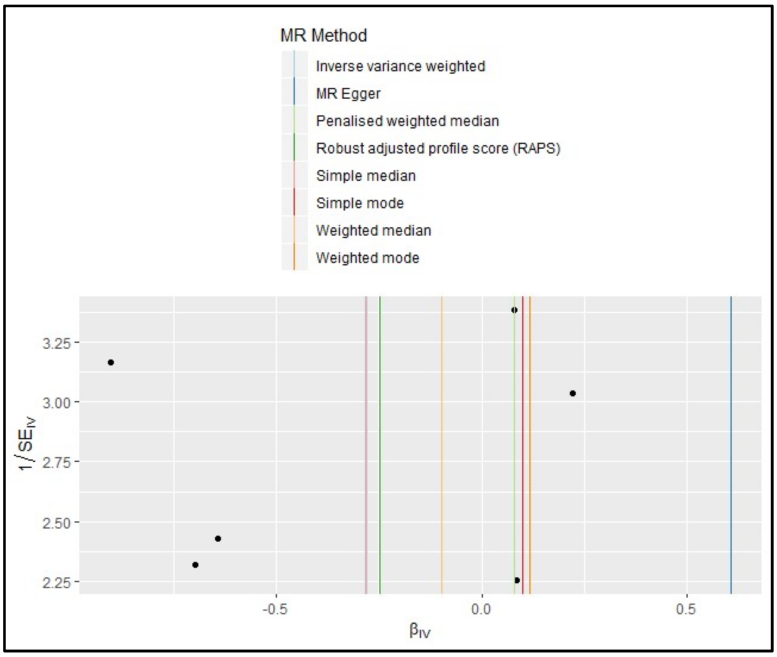

Figure S17. Funnel plot: Mendelian randomization (MR) analyses with leptin levels (European ancestry, additive model) (Yaghootkar et al., 2020) as exposure and anorexia nervosa (Watson et al., 2019) as outcome. BMI-associated SNP rs1121980 was excluded.

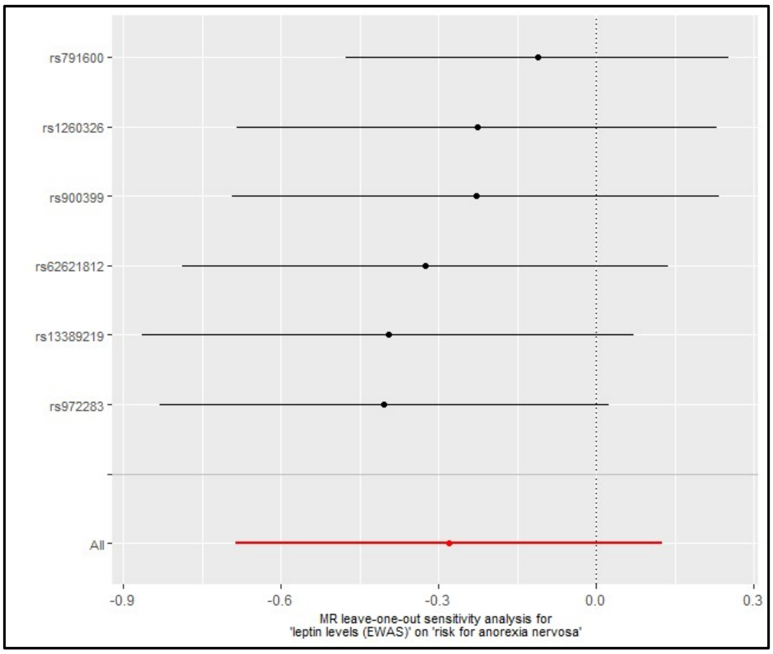

Figure S18. Leave out analyses using IVW method: Mendelian randomization (MR) analyses with leptin levels (European ancestry, additive model) (Yaghootkar et al., 2020) as exposure and risk for anorexia nervosa (Watson et al., 2019) as outcome. BMI-associated SNP rs1121980 was excluded.

Table S4. Single nucleotide polymorphisms (SNPs) associated with leptin levels unadjusted for BMI in females (European ancestry, additive model, ng/ml (rank based inverse normal transformation)) (Yaghootkar et al., 2020) and their association with the risk for anorexia nervosa (OR is transformed to beta) (Watson et al., 2019)

		Leptin levels in females							Anorexia nervosa				
SNP	Gene	EA	OA	EAF	b	se	p	F	EA	OA	b	se	p
rs1121980	*FTO*	A	G	0.431	0.044	0.009	6.2E-07	23.90	A	G	0.016	0.0135	0.2379
rs1260326	*GCKR*	C	T	0.606	0.040	0.009	6.0E-06	19.75	C	T	-0.022	0.0138	0.1057
rs13389219	*COBLL1*	T	C	0.392	0.038	0.009	6.1E-05	17.83	T	C	0.004	0.0136	0.7920
rs3799260	*KLHL31*	T	C	0.818	-0.043	0.011	8.9E-05	15.28	T	C	0.004	0.0171	0.8049
rs62621812	*ZNF800*	A	G	0.032	-0.103	0.024	2.1E-05	18.42	A	G	-0.008	0.0435	0.8495
rs791600	*LEP*	A	G	0.413	-0.054	0.009	8.3E-09	36.00	A	G	0.039	0.0136	0.0043
rs900399	*CCNL1*	G	A	0.397	-0.048	0.009	2.5E-07	28.44	G	A	0.021	0.0136	0.1184
rs972283	*KLF14*	G	A	0.521	-0.048	0.009	1.6E-08	28.44	G	A	-0.009	0.0135	0.5017

EA: effect allele; OA: other allele; EAF: effect allele frequency; b: effect size of EA; se: standard error; p: p-value; F: F-statistics

Table S5. Results of single SNP Mendelian randomization (MR) analyses and the overall causal effect of unadjusted leptin levels in females (European ancestry, additive model) (Yaghootkar et al., 2020) on the risk of anorexia nervosa (Watson et al., 2019) calculated using different methods. The beta estimates the change in risk for AN (OR is transformed to beta) per change of 1 SD in leptin level (ng/ml (rank based inverse normal transformation)).

SNP	b	se	p	Lower 95% CI	Upper 95% CI
rs1121980	0.361	0.307	0.239		
rs1260326	-0.557	0.345	0.106		
rs13389219	0.095	0.358	0.791		
rs3799260	-0.098	0.398	0.806		
rs62621812	0.081	0.422	0.849		
**rs791600**	**-0.720**	**0.252**	**0.004**		
rs900399	-0.442	0.283	0.119		
rs972283	0.190	0.281	0.500		
MR Egger	-0.428	0.874	0.642	-2.140	1.284
Inverse variance weighted	-0.185	0.150	0.217	-0.479	0.108
Simple median	-0.009	0.184	0.963	-0.369	0.351
Weighted median	-0.036	0.175	0.837	-0.379	0.307
Penalised weighted median	0.082	0.175	0.640	-0.262	0.426
Simple mode	0.104	0.327	0.760	-0.536	0.744
Weighted mode	0.147	0.380	0.710	-0.597	0.892
Robust adjusted profile score (RAPS)	-0.162	0.164	0.325	-0.484	0.161

Horizontal pleiotropy: Eggers intercept=0.0116; se=0.041: p=0.787; MR PRESSO: no outlier
Heterogeneity test: MR Egger Q(6)=12.34; p=0.055; IVW: Q(7)=12.50; p=0.085
b: effect size; se: standard error; p: p-value

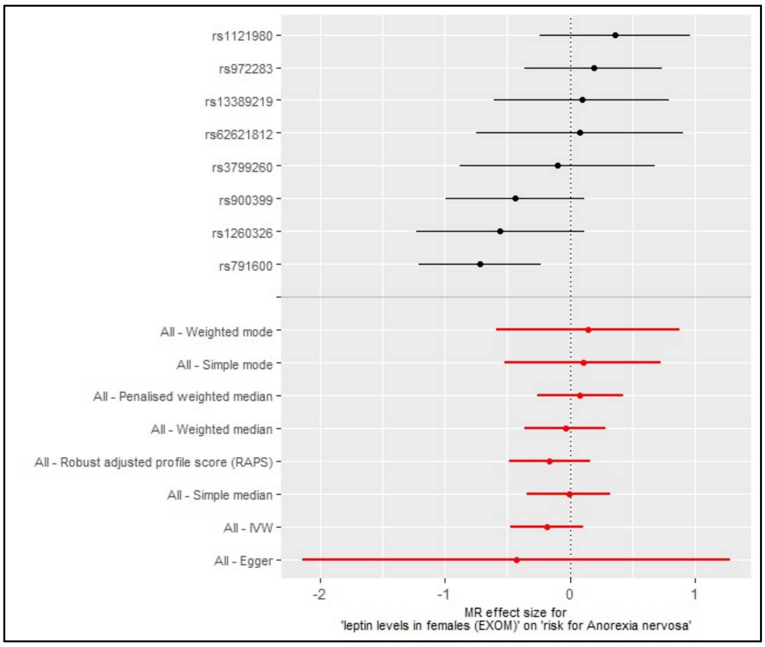

Figure S19. Results of the single and multi-SNP analyses for the SNP effect of leptin levels in females (Yaghootkar et al., 2020) on risk for anorexia nervosa (Watson et al., 2019). The black lines visualize the results of single SNP analyses; the red lines visualize the results of the multi SNP analysis

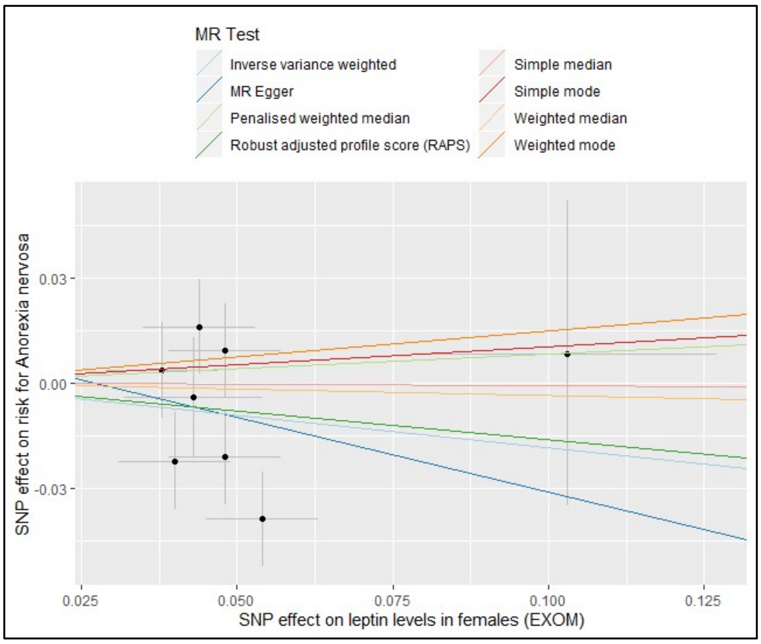

Figure S20. Scatter plots of genetic associations with leptin levels in females (European ancestry, additive model) (Yaghootkar et al., 2020) against risk for anorexia nervosa (Watson et al., 2019) using different Mendelian randomization (MR) methods. The slopes of each line represent the causal association for each method.

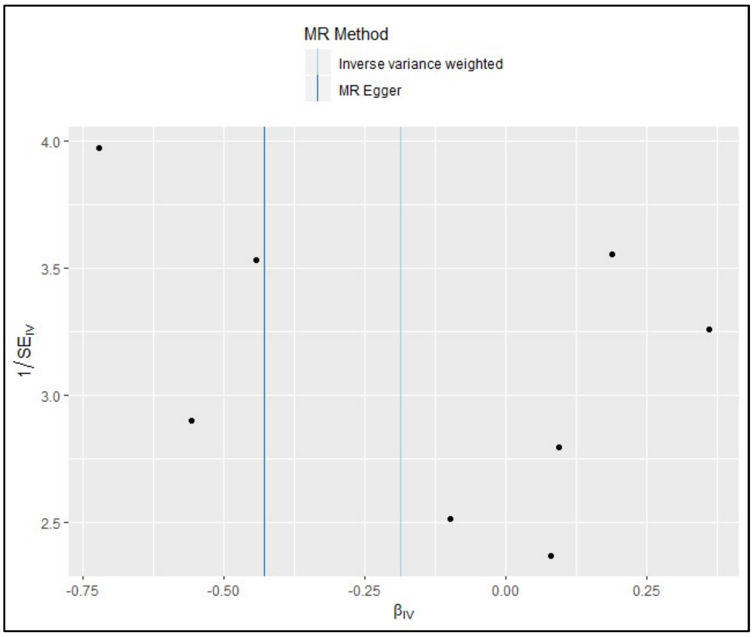

Figure S21. Funnel plot: Mendelian randomization (MR) analyses with leptin levels in female (European ancestry, additive model) (Yaghootkar et al., 2020) as exposure and anorexia nervosa (Watson et al., 2019) as outcome

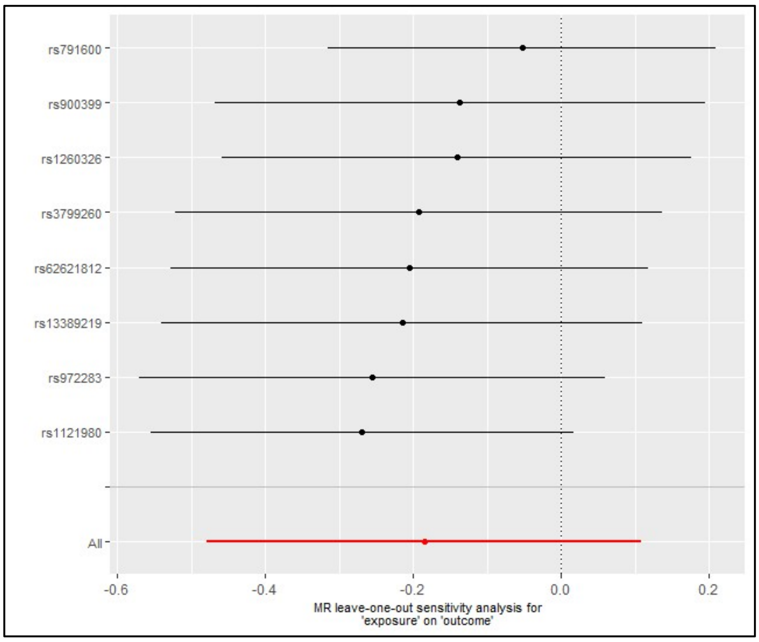

Figure S22. Leave out analyses using IVW method: Mendelian randomization (MR) analyses with leptin levels in females (European ancestry, additive model) (Yaghootkar et al., 2020) as exposure and risk for anorexia nervosa (Watson et al., 2019) as outcome

Table S6. Single nucleotide polymorphisms (SNPs) associated with leptin levels adjusted for BMI in females (European ancestry, additive model, ng/ml (rank based inverse normal transformation)) (Yaghootkar et al., 2020)

SNP	Nearest Gene	Leptin levels adjusted for BMI in females					
		EA	OA	EAF	b	SE	p
rs1121980	FTO	A	G	0.431	-0.009	0.009	3.20E-01
rs2340550	ACTL9	G	A	0.685	0.008	0.009	4.10E-01
rs1260326	GCKR	C	T	0.606	0.057	0.009	9.40E-11
rs13389219	COBLL1	T	C	0.392	0.055	0.009	3.80E-09
rs3799260	KLHL31	T	C	0.818	-0.057	0.011	2.20E-07
rs62621812	ZNF800	A	G	0.032	-0.126	0.024	1.90E-07
rs791600	LEP	A	G	0.413	-0.08	0.009	2.90E-17
rs900399	CCNL1	G	A	0.398	-0.058	0.009	3.40E-10
rs972283	KLF14	G	A	0.521	-0.066	0.009	1.30E-14

EA: effect allele; OA: other allele; EAF: effect allele frequency; b: effect size of EA; se: standard error; p: p-value

Table S7. Results of single SNP Mendelian randomization analyses and the overall causal effect of leptin levels in females (European ancestry, additive model) (Yaghootkar et al., 2020) on the risk of anorexia nervosa (Watson et al., 2019) calculated using different methods. The SNP rs1121980 (*FTO*) was excluded. The beta estimates the change in risk for AN (OR is transformed to beta) per change of 1 SD in leptin level (ng/ml (rank based inverse normal transformation)).

SNP	b	se	p	Lower 95% CI	Upper 95% CI
rs1260326	-0.557	0.345	0.106		
rs13389219	0.095	0.358	0.791		
rs3799260	-0.098	0.398	0.806		
rs62621812	0.081	0.422	0.849		
**rs791600 (LEP)**	**-0.720**	**0.252**	**0.004**		
rs900399	-0.442	0.283	0.119		
rs972283	0.190	0.281	0.500		
MR Egger	-0.317	0.814	0.713	-1.912	1.278
Inverse variance weighted	-0.269	0.146	0.066	-0.556	0.017
Simple median	-0.098	0.202	0.629	-0.494	0.298
Weighted median	-0.212	0.172	0.216	-0.549	0.124
Penalised weighted median	-0.212	0.173	0.220	-0.552	0.127
Simple mode	0.056	0.339	0.875	-0.608	0.719
Weighted mode	0.082	0.358	0.827	-0.620	0.784
Robust adjusted profile score (RAPS)	-0.256	0.162	0.115	-0.574	0.063

Horizontal pleiotropy: Eggers intercept=0.002; SE=0.038; p=0.954; MR PRESSO: no outlier
Heterogeneity: based on MR Egger Q(5)=8.837; p=0.116; based on IVW Q(6)=8.843; p=0.183
b: effect size; se: standard error; p: p-value

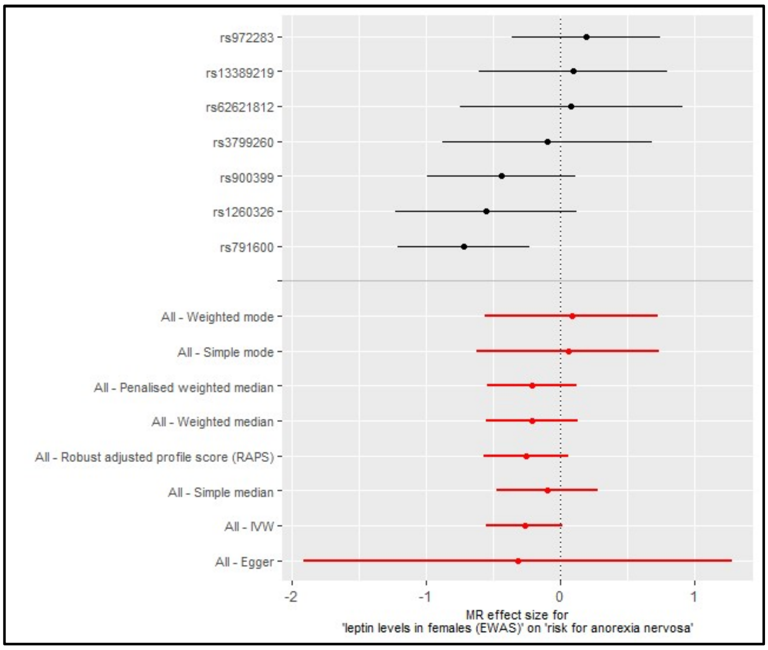

Figure S23. Results of the single and multi-SNP analyses for the SNP effect of leptin levels in females (Yaghootkar et al., 2020) on risk for Anorexia Nervosa (Watson et al., 2019). The SNP rs1121980 (*FTO*) was excluded. The black lines visualize the results of single SNP analyses; the red lines visualize the results of the multi SNP analysis

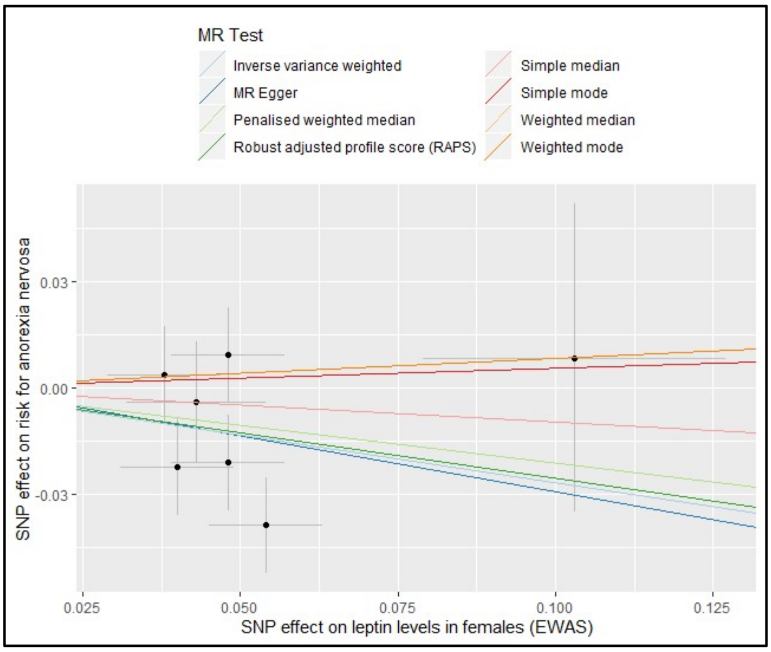

Figure S24. Scatter plots of genetic associations with leptin levels in females (European ancestry, additive model) (Yaghootkar et al., 2020) against risk for Anorexia Nervosa (Watson et al., 2019) using different MR methods. The SNP rs1121980 (*FTO*) was excluded. The slopes of each line represent the causal association for each method.

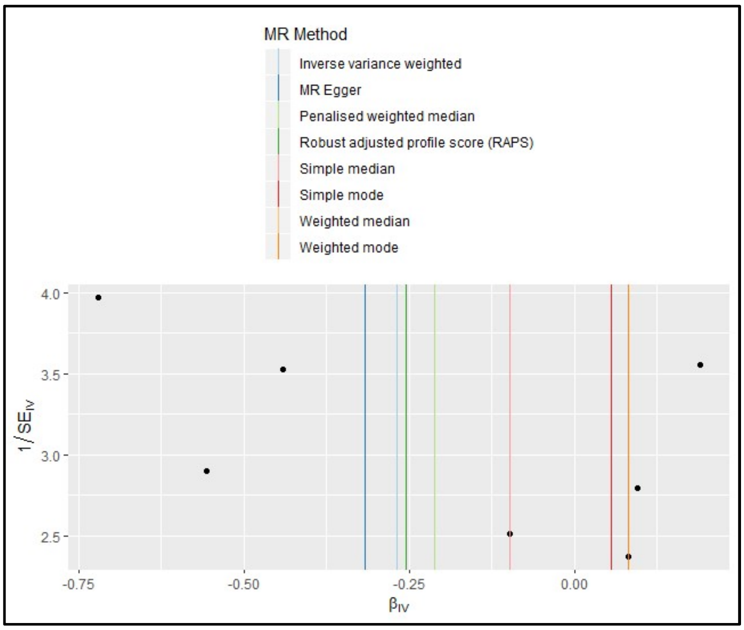

Figure S25. Funnel plot: Mendelian randomization (MR) analyses with leptin levels in females (European ancestry, additive model) (Yaghootkar et al., 2020) as exposure and anorexia nervosa (Watson et al., 2019) as outcome. The SNP rs1121980 (*FTO*) was excluded.

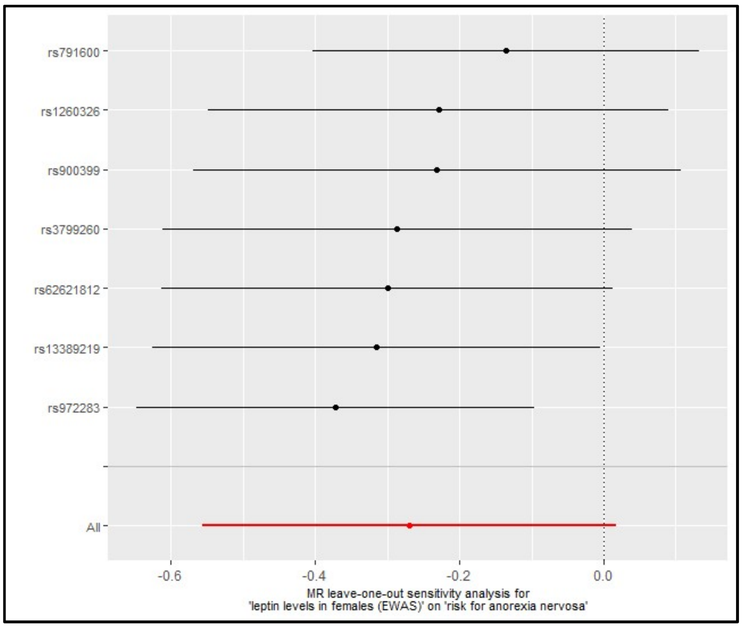

Figure S26. Leave out analyses using IVW method: Mendelian randomization (MR) analyses with leptin levels in females (European ancestry, additive model) (Yaghootkar et al., 2020) as exposure and risk for anorexia nervosa (Watson et al., 2019) as outcome. The SNP rs1121980 (*FTO*) was excluded.

Reverse MR: Effekt of anorexia nervosa on leptin levels

Table S8. Significant single nucleotide polymorphisms (SNPs) on risk for anorexia nervosa (AN) (OR is transformed to beta) (Watson et al., 2019) and their association with leptin levels unadjusted for BMI (µg/ml (log-transformation)) (Kilpeläinen et al., 2016). Five SNPs are replaced with proxy SNPs because they were not available in the GWAS for leptin levels.

SNP in GWAS	SNP in MR	Dis-tance		Anorexia nervosa						Leptin levels unadjusted				
			R^2	EA	OA	b	se	p	F	EA	OA	b	se	p
rs10747478	rs11165643	22642	1.00	T	C	-0.0745	0.0135	3.465e-08	30.45	T	C	0.0140	0.0063	0.027
rs13100344	rs7619675	-11148	0.86	A	C	0.0746	0.0137	5.225e-08	29.65	A	C	-0.0002	0.0068	0.978
rs2008387	rs2008387	0	1.00	A	G	0.0815	0.0145	1.725e-08	31.59	A	G	-0.0039	0.0069	0.569
rs2287348	rs2287348	0	1.00	T	C	0.1044	0.0179	5.619e-09	34.02	T	C	-0.0078	0.0083	0.346
rs370838138	rs6872919	4595	0.98	A	C	-0.0743	0.0135	4.053e-08	30.29	A	C	0.0056	0.0063	0.373
rs6589488	rs17649730	-55527	0.85	T	C	0.1214	0.0196	5.507e-10	38.36	T	C	-0.0016	0.0096	0.868
rs9821797	rs12107418	-28466	0.99	A	G	-0.1538	0.0201	1.903e-14	58.55	A	G	0.0041	0.0098	0.675
rs9874207	rs9874207	0	1.00	T	C	-0.0813	0.0145	2.048e-08	31.44	T	C	-0.0001	0.0069	0.991

R^2 : linkage disequilibrium between SNP in GWAS und proxy SNP
Distance: distance between the SNP in GWAS and the SNP in MR
EA: effect allele; OA: other allele; b: effect size of EA; se: standard error; p: p-value; F: F-statistics

Table S9. Results of single SNP Mendelian randomization (MR) analyses and the overall causal effect of risk for anorexia nervosa (AN) (Watson et al., 2019) on leptin levels unadjusted for BMI (Kilpeläinen et al., 2016) calculated using different MR methods.

SNP	b	se	p	Lower 95% CI	Upper 95% CI
**rs11165643**	**-0.187**	**0.084**	**0.026**		
rs12107418	-0.027	0.064	0.676		
rs17649730	-0.013	0.079	0.868		
rs2008387	-0.048	0.085	0.572		
rs2287348	-0.075	0.079	0.347		
rs6872919	-0.075	0.085	0.374		
rs7619675	-0.003	0.091	0.977		
rs9874207	0.001	0.085	0.988		
MR Egger	0.028	0.115	0.814	-0.197	0.253
Inverse variance weighted	-0.052	0.028	0.069	-0.107	0.004
Simple median	-0.037	0.035	0.281	-0.105	0.030
Weighted median	-0.034	0.036	0.352	-0.105	0.037
Penalised weighted median	-0.034	0.035	0.328	-0.102	0.034
Simple mode	-0.025	0.050	0.628	-0.123	0.073
Weighted mode	-0.027	0.051	0.615	-0.126	0.073
Robust adjusted profile score (RAPS)	-0.050	0.030	0.096	-0.108	0.009

Horizontal pleiotropy: Eggers intercept -0.008, SE=0.010, p=0.499; MR PRESSO no outlier
Heterogeneity test based on MR Egger Q(df=6)=3.21, p =0.769; based on IVW Q(df=7)=3.82, p =0.800
b: effect size; se: standard error; p: p-value; CI: confidence interval

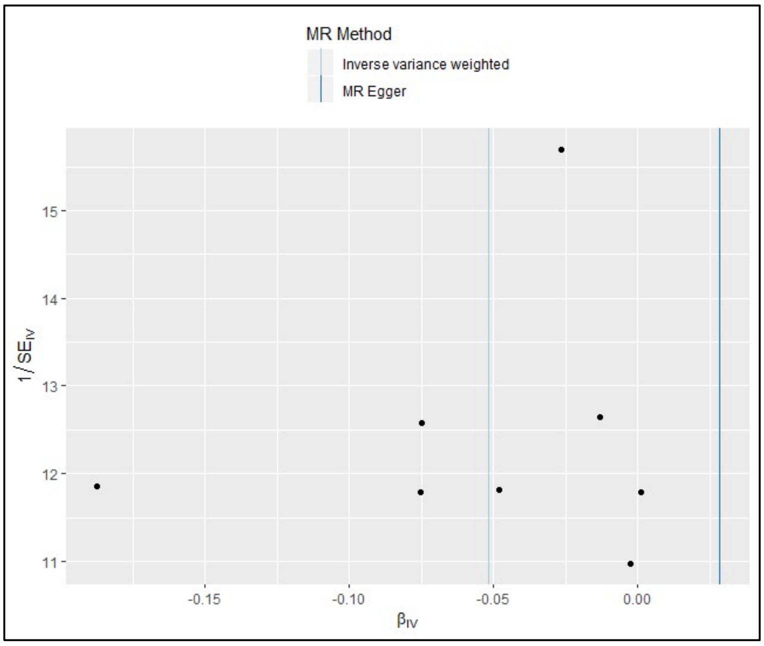

Figure S27. Funnel plot: Mendelian randomization (MR) analyses with risk for anorexia nervosa (Watson et al., 2019) as exposure and leptin levels (Kilpeläinen et al., 2016) as outcome

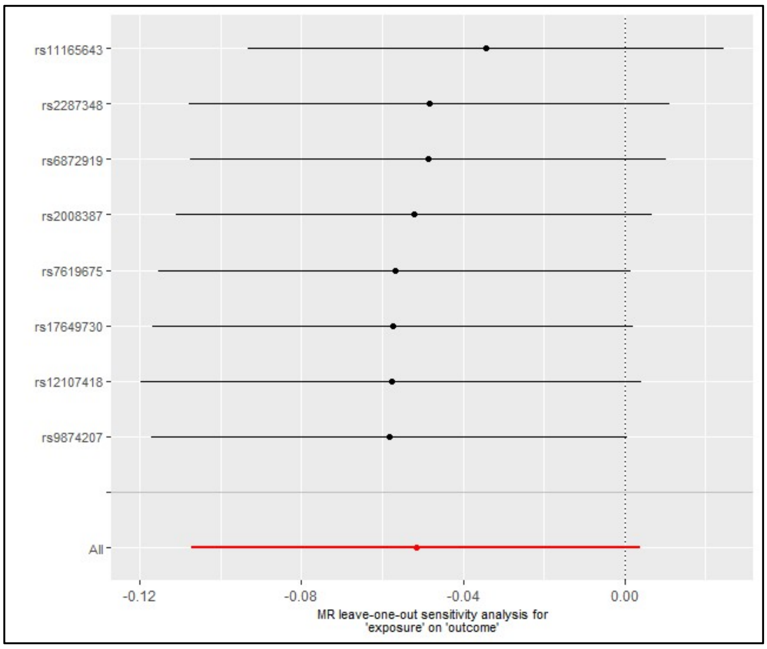

Figure S28. Leave out analyses using IVW method: Mendelian randomization (MR) analyses with anorexia nervosa as exposure (Watson et al., 2019) and leptin levels (Kilpeläinen et al., 2016) as outcome

References

Bulik-Sullivan, B. K., Loh, P. R., Finucane, H. K., Ripke, S., Yang, J., Schizophrenia Working Group of the Psychiatric Genomics, C., . . . Neale, B. M. (2015). LD Score regression distinguishes confounding from polygenicity in genome-wide association studies. *Nat Genet, 47*(3), 291-295. doi:10.1038/ng.3211

Kilpeläinen, T. O., Carli, J. F., Skowronski, A. A., Sun, Q., Kriebel, J., Feitosa, M. F., . . . Loos, R. J. (2016). Genome-wide meta-analysis uncovers novel loci influencing circulating leptin levels. *Nat Commun, 7*, 10494. doi:10.1038/ncomms10494

Watson, H. J., Yilmaz, Z., Thornton, L. M., Hubel, C., Coleman, J. R. I., Gaspar, H. A., . . . Bulik, C. M. (2019). Genome-wide association study identifies eight risk loci and implicates metabo-psychiatric origins for anorexia nervosa. *Nat Genet, 51*(8), 1207-1214. doi:10.1038/s41588-019-0439-2

Yaghootkar, H., Zhang, Y., C.N., S., .., & Kilpeläinen, T. O. (2020). Genetic studies of leptin concentrations implicate leptin in the regulation of early adiposity. *Diabetes, 69*(12), 2806-2818. doi:10.2337/db20-0070

Sensitivity analysis I

We performed two sensitivity analyses where we used SNPs detected in both GWAS and EWAS as IV. The LEP, GCKR, CCNL1, and FTO loci were detected in GWAS and EWAS, albeit with different SNPs. We did not include the SNPs associated with BMI (rs8043757, rs1121980).

1. GWAS by Kilpeläinen et al. (2016): SNPs rs10487505 (LEP), rs780093 (GCKR), rs900400 (CCNL1). Effect sizes are shown in Table 1 in manuscript.

Table 10. Results of single SNP Mendelian randomization (MR) analyses and the overall causal effect of leptin levels (Kilpeläinen et al., 2016) on risk for anorexia nervosa (AN) (Watson et al., 2019) calculated using different MR methods. Only SNPs (rs10487505 (LEP), rs780093 (GCKR), rs900400 (CCNL1)) were included which overlap with the loci of EWAS by Yaghootkar et al. (2020). The BMI-associated SNP rs8043757 were excluded. The beta estimates the change in risk for AN (OR is transformed to beta) per change of 1 unit of leptin concentration (log-transformed µg/ml).

MR method	b	se	p	Lower 95% CI	Upper 95% CI
MR Egger	1.034	2.034	0.701	-2.954	5.021
Inverse variance weighted	-0.877	0.276	**1.47E-03**	-1.418	-0.337
Simple median	-0.759	0.368	**0.039**	-1.481	-0.038
Weighted median	-0.746	0.333	**0.025**	-1.398	-0.094
Penalised weighted median	-0.746	0.342	**0.029**	-1.417	-0.075
Simple mode	-0.732	0.409	0.216	-1.533	0.070
Weighted mode	-0.734	0.378	0.192	-1.474	0.007
Robust adjusted profile score (RAPS)	-0.885	0.301	**3.25E-03**	-1.475	-0.296

MR PRESSO: not enough instrumental variables
Eggers intercept=-0.055, SE=0.058, p= 0.517;
Heterogeneity test based on MR Egger Q(df=1)=0.077, p =0.781); based on IVW (Q(df=2)=0.976, p =0.614

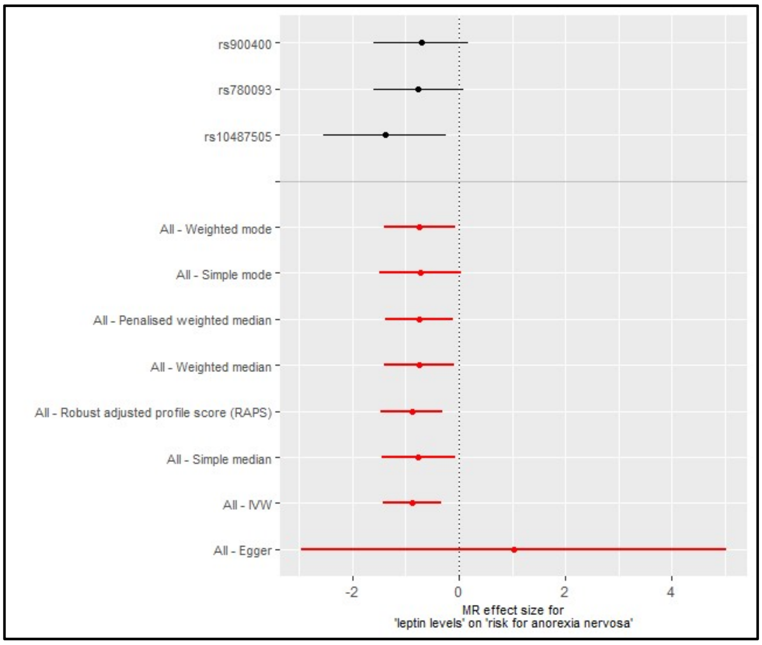

Figure S29. Results of the single and multiple-SNP Mendelian randomization (MR) analyses for effect of leptin levels (Kilpeläinen et al., 2016) on risk for anorexia nervosa (AN) (Watson et al., 2019). Only SNPs (rs10487505 (LEP), rs780093 (GCKR), rs900400 (CCNL1)) were included which overlap with the loci of EWAS by Yaghootkar et al. (2020). The black lines visualize the results of single SNP analyses; the red lines visualize the results of the multiple SNP analysis

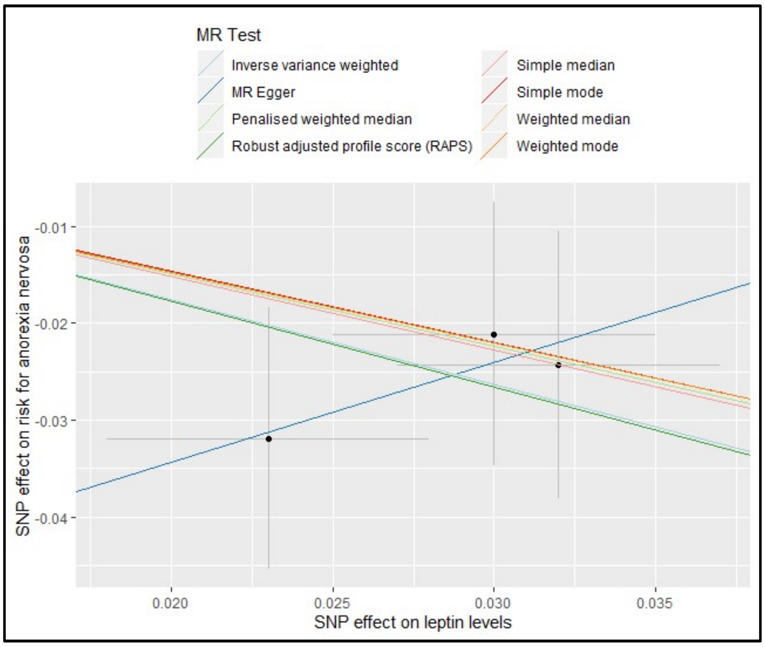

Figure 30. Scatter plots of genetic associations with leptin levels (Kilpeläinen et al., 2016) against risk for AN (Watson et al., 2019) using different Mendelian randomization (MR) methods. Only SNPs (rs10487505 (LEP), rs780093 (GCKR), rs900400 (CCNL1)) were included which overlap with the loci of EWAS by Yaghootkar et al. (2020). The slopes of each line represent the causal association for each method.

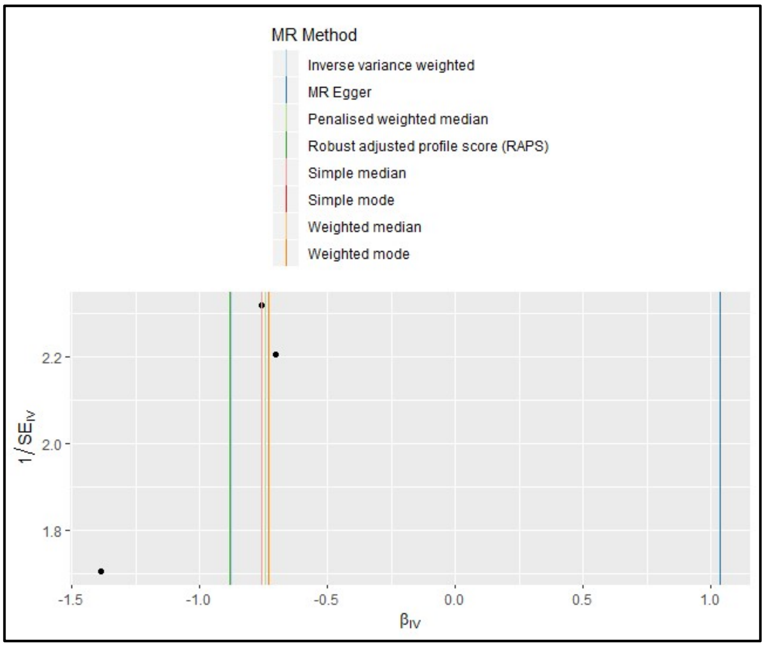

Figure 31. Funnel plot: Mendelian randomization (MR) analyses with leptin levels (Kilpeläinen et al., 2016) as exposure and risk for anorexia nervosa (AN) (Watson et al., 2019) as outcome. Only SNPs (rs10487505 (LEP), rs780093 (GCKR), rs900400 (CCNL1)) were included which overlap with the loci of EWAS by Yaghootkar et al. (2020).

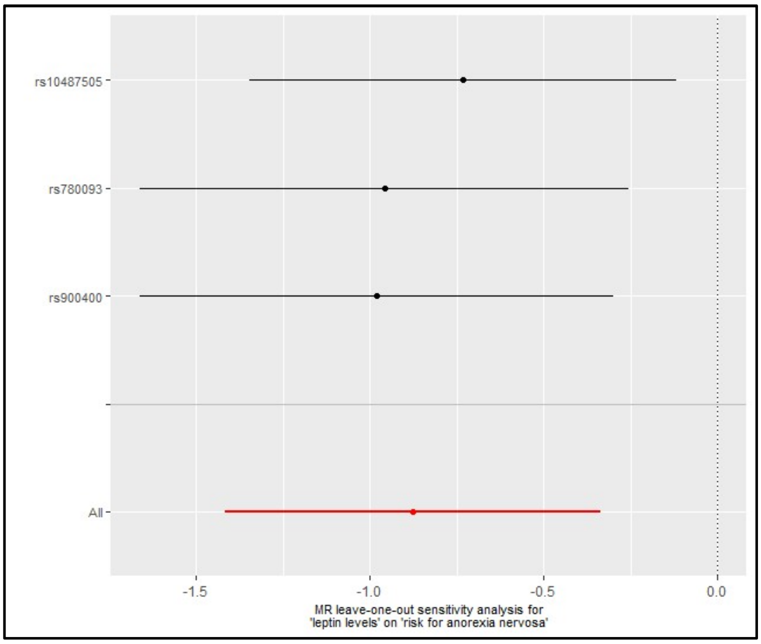

Figure S32. Leave out analyses using IVW method: Mendelian randomization (MR) analyses with leptin levels (Kilpeläinen et al., 2016) as exposure and risk for anorexia nervosa (Watson et al., 2019) as outcome. Only SNPs (rs10487505 (LEP), rs780093 (GCKR), rs900400 (CCNL1)) which overlap with the loci of EWAS by Yaghootkar et al. (2020) were included.

2. EWAS by Yaghootkar et al. (2020): SNPs rs791600 (*LEP*), rs1260326 (*GCKR*) and rs900399 (*CCNL1*). Effect sizes are shown in Table 3 in manuscript.

Table 11. Results of single SNP Mendelian randomization analyses and the overall causal effect of leptin levels (European ancestry, additive model) (Yaghootkar, Zhang, C.N., .., & Kilpeläinen, 2020) on the risk of anorexia nervosa (Watson et al., 2019) calculated using different methods. Only SNPs rs791600 (*LEP*), rs1260326 (*GCKR*) and rs900399 (*CCNL1*) which overlap with the loci detected by GWAS by Kilpeläinen et al. (2016) were included. The SNP rs1121980 (*FTO*) was excluded. The beta estimates the change in risk for AN (OR is transformed to beta) per change of 1 SD in leptin level (ng/ml (rank based inverse normal transformation)).

MR method	b	se	p	Lower 95% CI	Upper 95% CI
MR Egger	-1.616	1.586	0.494	-4.725	1.492
Inverse variance weighted	-0.779	0.217	**3.25E-04**	-1.205	-0.354
Simple median	-0.697	0.303	**0.021**	-1.290	-0.104
Weighted median	-0.743	0.265	**4.99E-03**	-1.262	-0.224
Penalised weighted median	-0.743	0.271	**6.07E-03**	-1.274	-0.212
Simple mode	-0.670	0.336	0.185	-1.329	-0.011
Weighted mode	-0.668	0.334	0.184	-1.323	-0.012
Robust adjusted profile score (RAPS)	-0.782	0.241	**1.16E-03**	-1.254	-0.310

MR PRESSO: not enough instrumental variables
Eggers intercept=-0.031, SE=0.058, p= 0.688;
Heterogeneity test based on MR Egger Q(df=1)=0.020, p =0.888); based on IVW (Q(df=2)=0.304, p =0.859

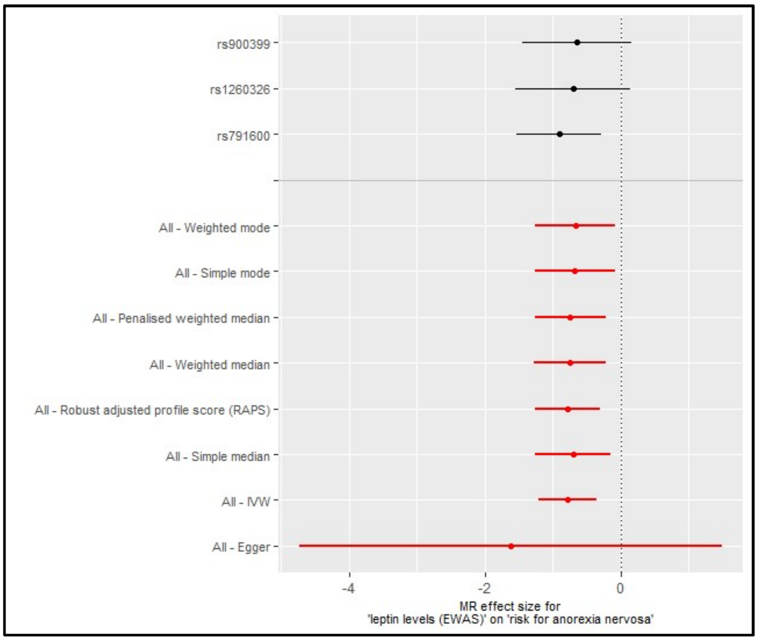

Figure S33. Results of the single and multi-SNP analyses for the SNP effect of leptin levels (Yaghootkar et al., 2020) on risk for Anorexia Nervosa (Watson et al., 2019). Only SNPs rs791600 (*LEP*), rs1260326 (*GCKR*) and rs900399 (*CCNL1*) which overlap with the loci detected by GWAS by Kilpeläinen et al. (2016) were included. The SNP rs1121980 (*FTO*) was excluded. The black lines visualize the results of single SNP analyses; the red lines visualize the results of the multi SNP analysis

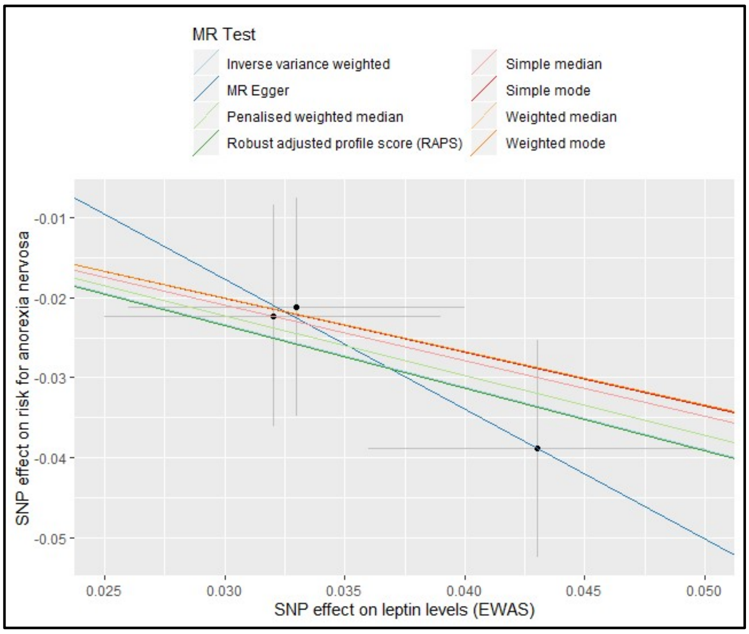

Figure S34. Scatter plots of genetic associations with leptin levels (European ancestry, additive model) (Yaghootkar et al., 2020) against risk for anorexia nervosa (Watson et al., 2019) using different Mendelian randomization (MR) methods. Only SNPs rs791600 (*LEP*), rs1260326 (*GCKR*) and rs900399 (*CCNL1*) which overlap with the loci detected by GWAS by Kilpeläinen et al. (2016) were included. The SNP rs1121980 (*FTO*) was excluded. The slopes of each line represent the causal association for each method.

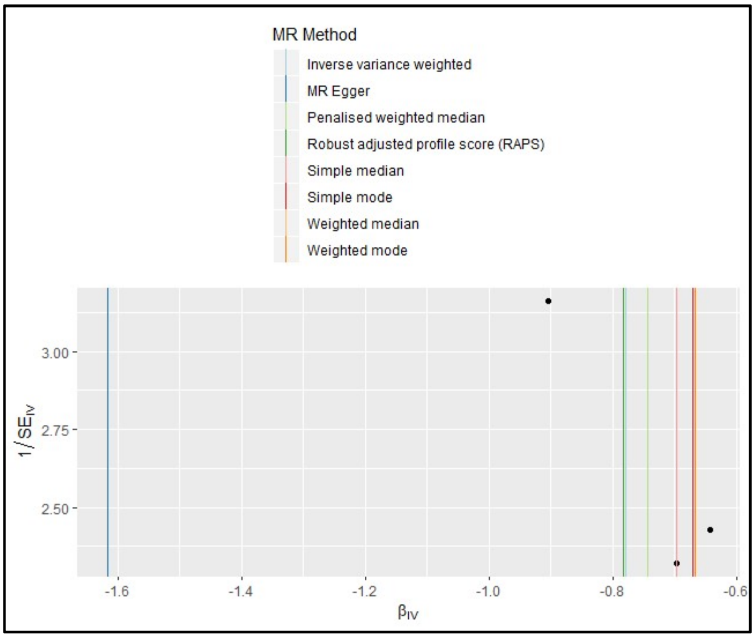

Figure S35. Funnel plot: Mendelian randomization (MR) analyses with leptin levels (European ancestry, additive model) (Yaghootkar et al., 2020) as exposure and anorexia nervosa (Watson et al., 2019) as outcome. Only SNPs rs791600 (*LEP*), rs1260326 (*GCKR*) and rs900399 (*CCNL1*) which overlap with the loci detected by GWAS by Kilpeläinen et al. (2016) were included. The SNP rs1121980 (*FTO*) was excluded.

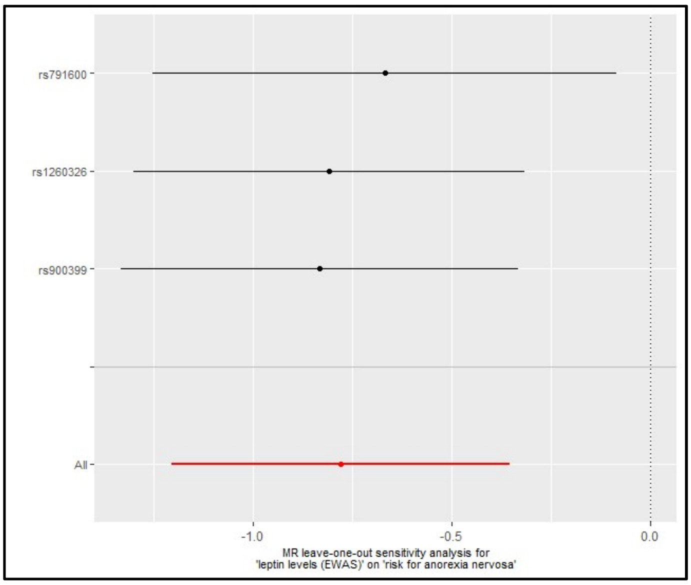

Figure S36. Leave out analyses using IVW method: Mendelian randomization (MR) analyses with leptin levels (European ancestry, additive model) (Yaghootkar et al., 2020) as exposure and risk for anorexia nervosa (Watson et al., 2019) as outcome. Only SNPs rs791600 (*LEP*), rs1260326 (*GCKR*) and rs900399 (*CCNL1*) which overlap with the loci detected by GWAS by Kilpeläinen et al. (2016) were included. The SNP rs1121980 (*FTO*) was excluded.

Sensitivity analysis II

We performed further sensitivity analyses where we used SNPs from EWAS (Yaghootkar et al., 2020) as IV, the effect sizes were driven von summary statistics by GWAS (Kilpeläinen et al., 2016). We did not include the SNP rs1121980 (*FTO*). The SNP rs62621812 and his proxy SNP rs111845633 (LD r^2=0.80) were not included in GWAS.

Table S12. Single nucleotide polymorphisms (SNPs) detected by EWAS (Yaghootkar et al., 2020) associated with leptin levels with effect sizes from GWAS (Kilpeläinen et al., 2016) (µg/ml (log-transformation)) and their association with the risk for anorexia nervosa (AN) (OR is transformed to beta) (Watson et al., 2019).

SNPs from EWAS	Chr	Nearest gene	EA	OA	Leptin levels (effect sizes from GWAS)			Anorexia nervosa		
					b	se	p	b	se	p
rs1260326	2	*GCKR*	T	C	-0.0323	0.0063	3.64E-07	0.0223	0.0138	0.1057
rs13389219	2	*COBLL1*	T	C	0.0276	0.0065	1.95E-05	0.0036	0.0136	0.792
rs62621812		*ZNF800*								
rs791600	7	*LEP*	A	G	-0.0277	0.0063	1.16E-05	0.0389	0.0136	0.004285
rs900399	3	*CCNL1*	A	G	0.0319	0.0065	1.09E-06	-0.021	0.0136	0.1184
rs972283	7	*KLF14*	A	G	0.0255	0.0067	1.44E-04	0.0091	0.0135	0.5017

Table S13. Results of single SNP Mendelian randomization (MR) analyses and the overall causal effect of leptin levels on risk for anorexia nervosa (AN) calculated using different MR methods. IV are SNPs from EWAS (Yaghootkar et al., 2020), the effect sizes are driven from summary statistics of GWAS (Kilpeläinen et al., 2016). The beta estimates the change in risk for AN (OR is transformed to beta) per change of 1 unit of leptin concentration (log-transformed µg/ml).

SNP	b	se	p	Lower 95% CI	Upper 95% CI
rs1260326	-0.690	0.427	0.106		
rs13389219	0.130	0.493	0.791		
rs791600	-1.404	0.491	**0.004**		
rs900399	-0.665	0.426	0.119		
rs972283	0.357	0.529	0.500		
MR Egger	-3.473	3.330	0.374	-10.000	3.054
Inverse variance weighted	-0.502	0.296	0.090	-1.082	0.078
Simple median	-0.665	0.308	**0.031**	-1.269	-0.060
Weighted median	-0.666	0.303	**0.028**	-1.260	-0.071
Penalised weighted median	-0.666	0.316	**0.035**	-1.286	-0.046
Simple mode	-0.608	0.464	0.260	-1.517	0.301
Weighted mode	-0.608	0.428	0.228	-1.446	0.230
MR RAPS	-0.526	0.315	0.095	-1.143	0.092

Horizontal pleiotropy: Eggers intercept -0.087, SE=0.097, p=0.436; MR PRESSO no outlier
Heterogeneity test based on MR Egger Q(df=3)=6.308, p =0.098; based on IVW Q(df=4)=7.995, p =0.092
b: effect size; se: standard error; p: p-value; CI: confidence interval

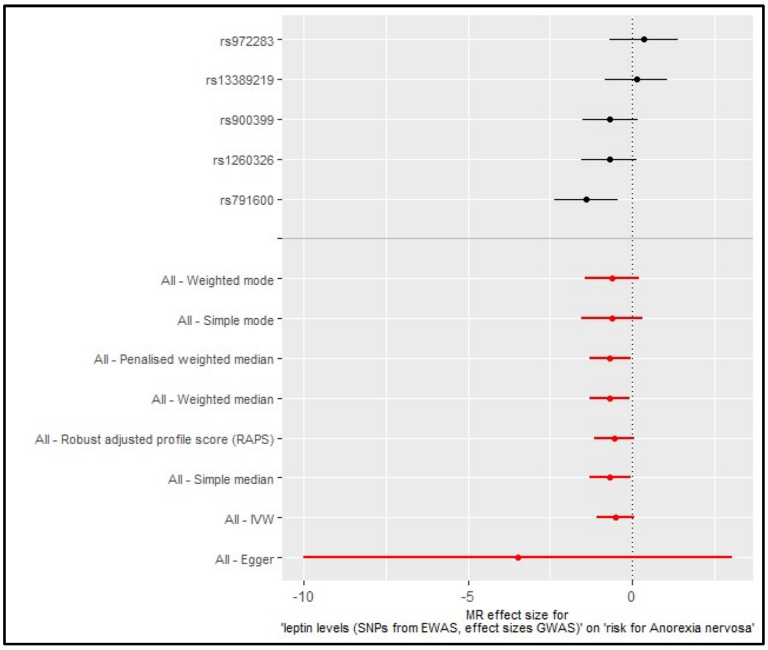

Figure S37. Results of single SNP Mendelian randomization (MR) analyses and the overall causal effect of leptin levels on risk for anorexia nervosa (AN) calculated using different MR methods. IV are SNPs from EWAS (Yaghootkar et al., 2020), the effect sizes are driven from summary statistics of GWAS (Kilpeläinen et al., 2016). The beta estimates the change in risk for AN (OR is transformed to beta) per change of 1 SD in leptin level (log-transformed µg/ml). The black lines visualize the results of single SNP analyses; the red lines visualize the results of the multi SNP analysis

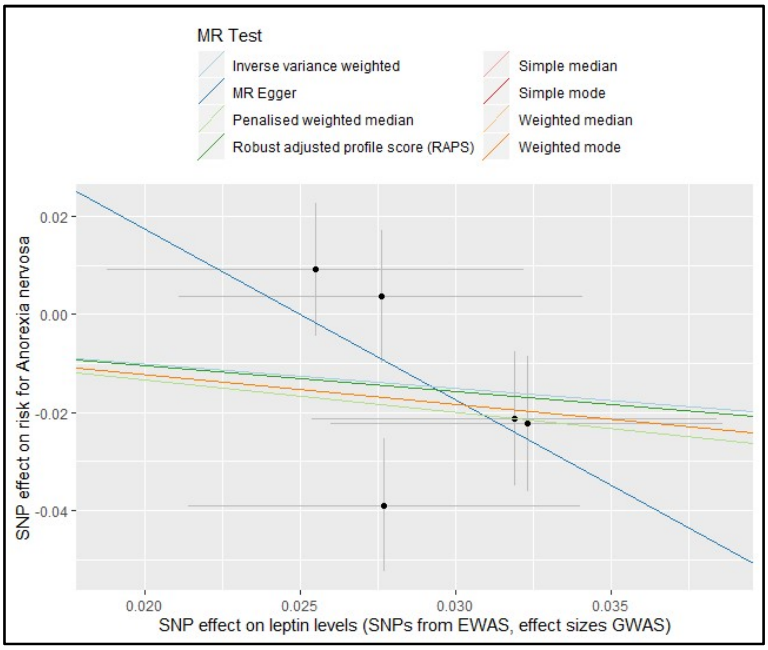

Figure S38. Scatter plots of genetic associations with leptin levels (Kilpeläinen et al., 2016) against risk for AN (Watson et al., 2019) using different Mendelian randomization (MR) methods. IV are SNPs from EWAS on leptin levels (Yaghootkar et al., 2020), the effect sizes are driven from summary statistics of GWAS (Kilpeläinen et al., 2016). The slopes of each line represent the causal association for each method.

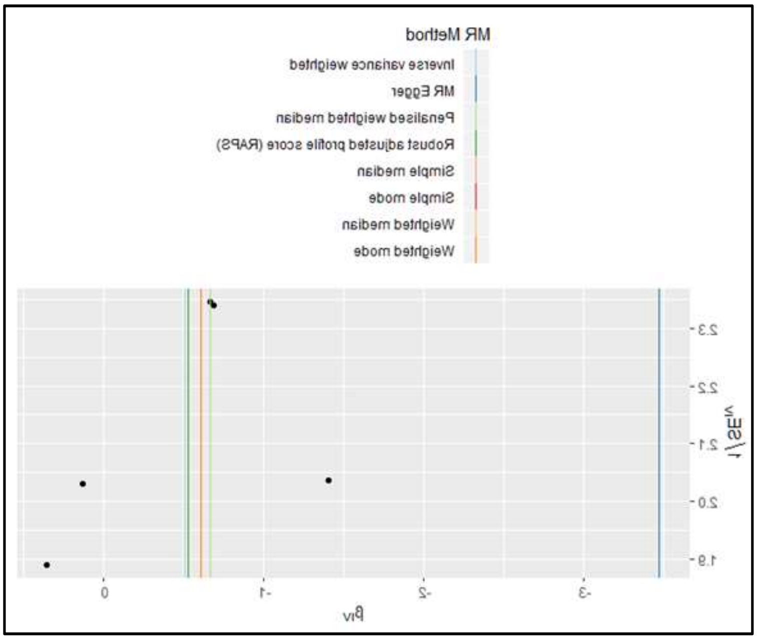

Figure S39. Funnel plot: Mendelian randomization (MR) analyses with leptin levels (Kilpeläinen et al., 2016) on risk for AN (Watson et al., 2019) using different Mendelian randomization (MR) methods. IV are SNPs from EWAS on leptin levels (Yaghootkar et al., 2020), the effect sizes are driven from summary statistics of GWAS (Kilpeläinen et al., 2016).

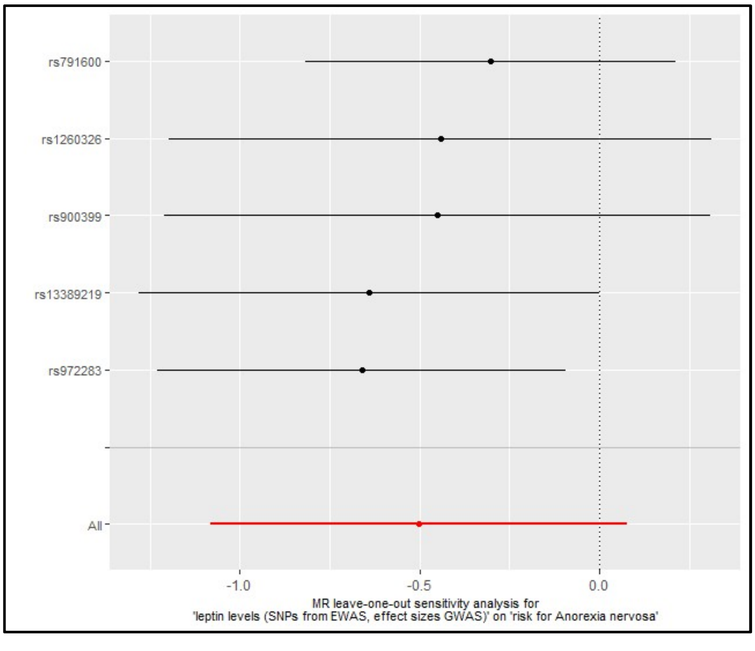

Figure S40. Leave out analyses using IVW method: Mendelian randomization (MR) analyses with leptin levels (Kilpeläinen et al., 2016) on risk for AN (Watson et al., 2019) using different Mendelian randomization (MR) methods. IV are SNPs from EWAS on leptin levels (Yaghootkar et al., 2020), the effect sizes are driven from summary statistics of GWAS (Kilpeläinen et al., 2016).

Kilpeläinen, T. O., Carli, J. F., Skowronski, A. A., Sun, Q., Kriebel, J., Feitosa, M. F., . . . Loos, R. J. (2016). Genome-wide meta-analysis uncovers novel loci influencing circulating leptin levels. *Nat Commun, 7*, 10494. doi:10.1038/ncomms10494

Watson, H. J., Yilmaz, Z., Thornton, L. M., Hubel, C., Coleman, J. R. I., Gaspar, H. A., . . . Bulik, C. M. (2019). Genome-wide association study identifies eight risk loci and implicates metabo-psychiatric origins for anorexia nervosa. *Nat Genet, 51*(8), 1207-1214. doi:10.1038/s41588-019-0439-2

Yaghootkar, H., Zhang, Y., C.N., S., .., & Kilpeläinen, T. O. (2020). Genetic studies of leptin concentrations implicate leptin in the regulation of early adiposity. *Diabetes, 69*(12), 2806-2818. doi:10.2337/db20-0070
